# Evolutionary Instability of Collateral Susceptibility Networks in Ciprofloxacin-Resistant Clinical Escherichia coli Strains

**DOI:** 10.1128/mbio.00441-22

**Published:** 2022-07-07

**Authors:** Vidar Sørum, Emma L. Øynes, Anna S. Møller, Klaus Harms, Ørjan Samuelsen, Nicole L. Podnecky, Pål J. Johnsen

**Affiliations:** a Department of Pharmacy, Faculty of Health Sciences, The University of Tromsø (UiT) The Arctic University of Norway, Tromsø, Norway; b Department of Biology, Saint Michael’s College, Colchester, Vermont, USA; c Norwegian National Advisory Unit on Detection of Antimicrobial Resistance, Department of Microbiology and Infection Control, University Hospital of North Norway, Tromsø, Norway; Emory University

**Keywords:** *Escherichia coli*, antimicrobial resistance, ciprofloxacin, collateral sensitivity, compensatory mutations

## Abstract

Collateral sensitivity and resistance occur when resistance development toward one antimicrobial either potentiates or deteriorates the effect of others. Previous reports on collateral effects on susceptibility focus on newly acquired resistance determinants and propose that novel treatment guidelines informed by collateral networks may reduce the evolution, selection, and spread of antimicrobial resistance. In this study, we investigate the evolutionary stability of collateral networks in five ciprofloxacin-resistant, clinical Escherichia coli strains. After 300 generations of experimental evolution without antimicrobials, we show complete fitness restoration in four of five genetic backgrounds and demonstrate evolutionary instability in collateral networks of newly acquired resistance determinants. We show that compensatory mutations reducing efflux expression are the main drivers destabilizing initial collateral networks and identify *rpoS* as a putative target for compensatory evolution. Our results add another layer of complexity to future predictions and clinical application of collateral networks.

## INTRODUCTION

The current discovery void in the development of novel antimicrobial agents is at the core of the global antimicrobial resistance crisis. We are at risk of running out of antimicrobials, and consequently, there is an urgent need to prolong the life span of existing, effective antimicrobial agents. One promising strategy is to exploit the concepts of collateral sensitivity, and its inverse collateral resistance, where resistance development toward specific antimicrobials modulates susceptibilities toward alternative agents (1). Collateral networks have been proposed for use in antimicrobial cycling protocols (2), sequential treatment regimens (3–5), and in combination therapies (6, 7) to limit, prevent, or reverse antimicrobial resistance evolution, selection, and spread (1, 8).

It is clear that the future application of collateral effects in antimicrobial resistance management depends on effective and accurate predictions informed by susceptibility testing. Early, seminal work has almost exclusively used emblematic laboratory strains and demonstrated the pervasiveness of collateral networks (2, 9, 10), elucidated mechanistic insights (9, 11), and/or proposed conceptual treatment strategies implementing collateral sensitivity (2, 12, 13). More recently, collateral sensitivity was shown to directly affect evolutionary trajectories of resistance in patients during treatment (14) and that principal contributors, including mechanisms of resistance, allow robust predictions of collateral susceptibility networks (4, 15, 16). Taken together, these reports allow for careful optimism for the future use of collateral networks as an antimicrobial resistance management strategy. However, with few exceptions (3, 17), the current body of literature rests on newly acquired resistance determinants upon which collateral networks are identified and characterized relative to a susceptible wild type (WT) in an optimal environment as the baseline. Thus, robust predictions of collateral networks are contingent on the evolutionary stability of the initial association between resistance determinants and bacterial hosts. A recent study demonstrated that Pseudomonas aeruginosa could not overcome collateral sensitivity by *de novo* mutations when challenged with antimicrobials, suggesting robust collateral networks for specific combinations of antimicrobials (3). However, the effect of coevolution between the bacterial host and its newly acquired antibiotic resistance determinants on the sign and magnitude of collateral networks is currently unknown.

Here, we asked if collateral networks were stable over 300 generations of experimental evolution in the absence of antimicrobial selection. We focus here on five different clinical Escherichia coli strains harboring newly acquired ciprofloxacin resistance due to combinations of drug target and efflux mutations. Evolved strains were characterized with respect to their collateral networks, relative fitness measurements, whole-genome sequencing, and gene expression analyses. We demonstrate the evolutionary instability of collateral networks after experimental evolution independent of resistance mechanisms and fitness compensatory mutations. These results introduce an additional layer of complexity with respect to accurate predictions of collateral networks in clinical application.

## RESULTS

### Collateral networks display evolutionary instability.

**FIG 1:**
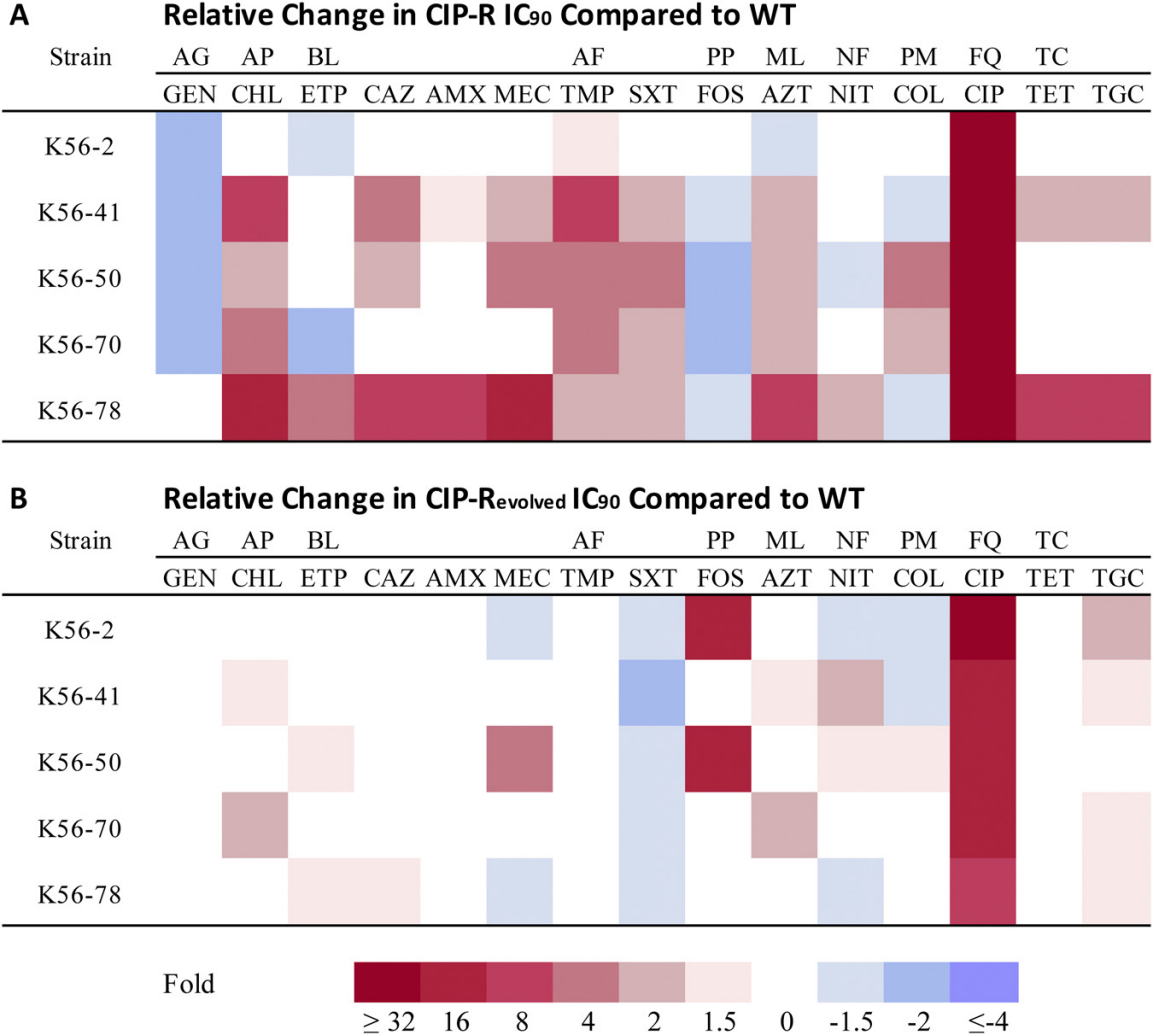
Collapsed collateral networks in ciprofloxacin-resistant mutants following experimental evolution (300 generations) in the absence of antimicrobial selection. (A) Heatmap showing the average fold change difference in the susceptibility of ciprofloxacin-resistant mutants compared with their respective WT tested toward a panel of 15 antimicrobials (data reported previously in reference 4). (B) Heatmap showing the average fold change difference in susceptibility of ciprofloxacin-resistant mutants compared with WT after 300 generations in the absence of antimicrobial selective pressure. The reported values (Fig. 1B) are the average of the three different parallel evolved populations for each strain. For a population-specific heatmap, see Fig. S1. Drug classes and their abbreviations are as follows: aminoglycoside (AG), amphenicol (AP), beta-lactam (BL), antifolate (AF), phosphonic (PP), macrolide (ML), nitrofuran (NF), polymyxin (PM), fluoroquinolone (FQ), and tetracycline (TC). Antimicrobials from left to right: gentamicin (GEN), chloramphenicol (CHL), ertapenem (ETP), ceftazidime (CAZ), amoxicillin (AMX), mecillinam (MEC), trimethoprim (TMP), trimethoprim-sulfamethoxazole (SXT), fosfomycin (FOS), azithromycin (AZT), nitrofurantoin (NIT), colistin (COL), ciprofloxacin (CIP), tetracycline (TET), and tigecycline (TGC).

We previously reported conserved collateral effects in a collection of genetically diverse clinical strains of E. coli isolated from urinary tract infections (UTIs) (4). From that study, we chose five laboratory-selected ciprofloxacin-resistant (CIP-R) E. coli strains (4) covering a diverse set of target and efflux mutations (Table 1). From each strain, three parallel populations (A, B, and C) and their corresponding WTs were used to initiate a total of 30 populations which we subjected to experimental evolution for 300 generations in the absence of antimicrobial selection (CIP-R_evolved_ and WT_evolved_, respectively). Susceptibility testing (90% inhibitory concentration [IC_90_] measurements) following experimental evolution revealed that the initial collateral effects (Fig. 1A) displayed by E. coli strains were largely lost (Fig. 1B), including conserved patterns of collateral sensitivity toward gentamicin and fosfomycin that were observed in four of five genetic backgrounds and the widespread efflux-mediated cross-resistance patterns (4). At the same time, ciprofloxacin resistance levels were reduced in populations founded by strains harboring combinations of *gyrA* and various efflux mutations, a phenomenon also observed by others (18). Given the strong linear correlation between fold changes in IC_90_ and MIC (6, 19), as well as absolute number comparisons (4), we conclude that the levels of ciprofloxacin resistance were reduced to just below the clinical breakpoint of 0.5 mg/L (20). Notably, ciprofloxacin resistance levels were still 14- to 20-fold higher than ancestral WT IC_90_. On the other hand, populations founded by the ancestral strain K56-2 CIP-R, which only harbored drug target mutations, maintained resistance levels above the clinical breakpoint after experimental evolution (average IC_90_, 7.55 mg/L). K56-2 CIP-R_evolved_ population A and K56-50 CIP-R_evolved_ population A both showed a marked increase of 17.3- and 21.3-fold, respectively, in fosfomycin resistance after evolution. This finding is likely due to the complex mutational landscape caused by mutations in the DNA mismatch repair genes *mutS* and *mutL* in these populations (see Table S1 in the supplemental material). We also observed a conserved sensitization toward trimethoprim-sulfamethoxazole (SXT). Investigating population-specific collateral effects identified several instances of evolutionary divergence between evolved populations of the same strain background toward the different antibiotics (see Fig. S1 in the supplemental material). For dose response curves see Fig. S2 in the supplemental material.

**TABLE 1:** Genes with identified initial resistance mutations and additional putative compensatory mutations in clinical E. coli isolates after 300 generations in the absence of antimicrobials

Strain background	Gene	Gene function	Putative CIP-R/compensatory (mechanism or pathway)
K56-2 CIP-R	*gyrA*	DNA gyrase (type II topoisomerase), subunit A	Drug target modification
	*gyrA*	DNA gyrase (type II topoisomerase), subunit A	Drug target modification
	*parC*	DNA topoisomerase IV, subunit A	Drug target modification
K56-2 CIP-R_evolved_	*fyuA*	Siderophore yersiniabactin receptor	Regulator
	*rpoS*	RNA polymerase sigma factor	Regulator/stress response
K56-41 CIP-R	*gyrA*	DNA gyrase (type II topoisomerase), subunit A	Drug target modification
	*rpoC*	RNA polymerase, beta prime subunit	Increased drug efflux (MdtK)
K56-41 CIP-R_evolved_	*rpoC* ^*a*^	RNA polymerase, beta prime subunit	Decreased drug efflux
	*rpoB*	RNA polymerase, beta subunit	Decreased drug efflux
K56-50 CIP-R	*gyrA*	DNA gyrase (type II topoisomerase), subunit A	Drug target modification
	*rpoC*	RNA polymerase, beta prime subunit	Increased drug efflux (MdtK)
	*soxR*	redox-sensitive transcriptional activator of *soxS*	Increased drug efflux (AcrAB-TolC)
K56-50 CIP-R_evolved_	*soxS* ^*b*^ ^*c*^	Transcriptional activator of the superoxide response regulon	Decreased drug efflux
	*rpoC* ^*d*^	RNA polymerase, beta prime subunit	Decreased drug efflux (MdtK)
	*rpoB*	RNA polymerase, beta subunit	Decreased drug efflux (MdtK)
	*rpoA* ^*a*^ ^*c*^	RNA polymerase alpha subunit	Regulator/decreased drug efflux
K56-70 CIP-R	*gyrA*	DNA gyrase (type II topoisomerase), subunit A	Drug target modification
	*rpoB* ^*d*^	RNA polymerase, beta subunit	Increased drug efflux (MdtK)
	*marR*	Transcriptional repressor	Increased drug efflux (AcrAB-TolC)
K56-70 CIP-R_evolved_	*rpoB*	RNA polymerase, beta subunit	Decreased drug efflux (MdtK)
	*fyuA*	Siderophore yersiniabactin receptor	Regulator
K56-78 CIP-R	*gyrA*	DNA gyrase (type II topoisomerase), subunit A	Drug target modification
	Intergenic region (28 upstream of *rpoB*)	RNA polymerase, beta subunit	Increased drug efflux (MdtK)
	*rpoB*	RNA polymerase, beta subunit	Increased drug efflux (MdtK)
	*marR*	Transcriptional repressor	Increased drug efflux (AcrAB-TolC)
	*acrR*	Transcriptional repressor	Increased drug efflux (AcrAB-TolC)
K56-78 CIP-R_evolved_	*dnaQ* ^*c*^	DNA polymerase III epsilon subunit	DNA repair/stability
	*dksA*	RNA polymerase-binding transcription factor	Regulator

a Mutation in the same gene as the CIP-R ancestor. CIP-R mutations not mentioned in the CIP-R_evolved_ lines are still present.

b Found in population with *mutS/L.*

c Not parallel evolution defined as genetic alterations to the same genetic target between different populations and/or strains.

d Mutation to the same nucleotide as CIP-R ancestor resulting in a changed amino acid.

10.1128/mbio.00441-22.1 TABLE S1 Full list of ancestral and evolved mutations. Download Table S1, XLSX file, 0.05 MB. Copyright © 2022 Sørum et al. 2022 Sørum et al. https://creativecommons.org/licenses/by/4.0/ This content is distributed under the terms of the Creative Commons Attribution 4.0 International license.

10.1128/mbio.00441-22.5 FIG S1 Heatmap of population-specific changes in IC_90_. Download FIG S1, PDF file, 0.2 MB. Copyright © 2022 Sørum et al. 2022 Sørum et al. https://creativecommons.org/licenses/by/4.0/ This content is distributed under the terms of the Creative Commons Attribution 4.0 International license.

10.1128/mbio.00441-22.6 FIG S2 Dose response curves. Download FIG S2, PDF file, 1.5 MB. Copyright © 2022 Sørum et al. 2022 Sørum et al. https://creativecommons.org/licenses/by/4.0/ This content is distributed under the terms of the Creative Commons Attribution 4.0 International license.

### Collapse of collateral networks is linked to compensatory evolution.

**FIG 2:**
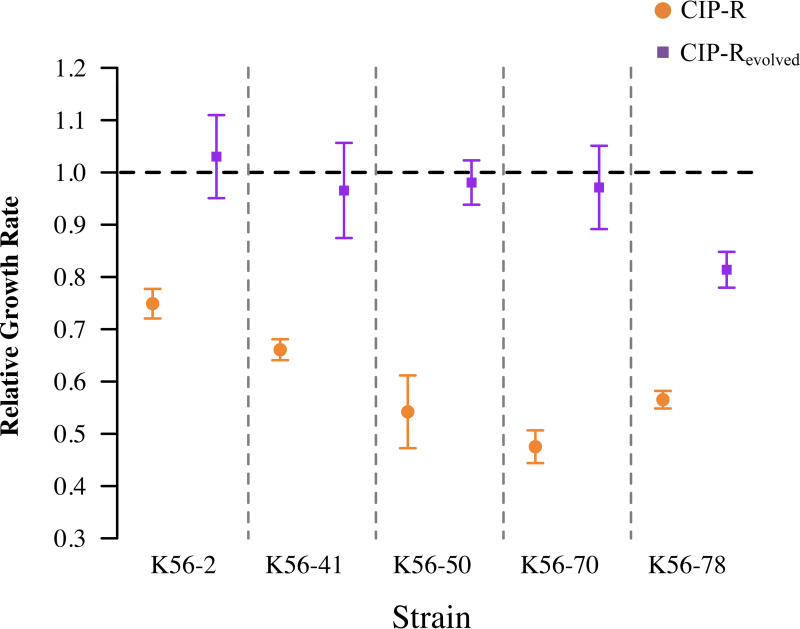
Relative growth rate of ciprofloxacin-resistant mutants compared with their respective WT before* (orange) and after (purple) experimental evolution. Evolved ciprofloxacin-resistant populations were compared with evolved WT populations to ameliorate fitness effects caused by medium adaptation. Values below 1 (horizontal dashed line) denote a decreased growth rate in resistant mutants compared with their respective WT. We observed a reduction in the cost of ciprofloxacin resistance after evolution in all tested strains (*P* < 0.05). However, K56-78 CIP-R_evolved_ was the only strain that still displayed a significantly reduced growth rate compared with WT after evolution (*P* < 0.0001). Data points represent the average value, and error bars denote the 95% confidence interval, *n* = 9. *, Data reported previously in reference 4.

We reported previously that the fitness cost of resistance was a principal contributor to collateral effects (4). In this study, we hypothesized that the observed evolutionary instability of collateral effects (Fig. 1) was driven by compensatory evolution. To test this hypothesis, we used relative growth rates as a proxy for fitness before and after experimental evolution. All evolved ciprofloxacin-resistant mutants displayed significant ameliorations of initial fitness costs relative to WT controls (all *P* < 0.05) (Fig. 2), which were also evolved for 300 generations to control for putative effects of medium adaptations. In strains harboring combinations of *gyrA* and various efflux mutations, relative fitness was fully restored (all *P* > 0.97) (Fig. 2, Table S2), except for that of K56-78 CIP-R_evolved_ (*P* < 0.0001) (Fig. 2, Table S2). K56-78 CIP-R had the most complex resistance mutation profile affecting two different efflux pumps (MdtK and AcrAB-TolC) in addition to *gyrA* (Table 1).

10.1128/mbio.00441-22.2 TABLE S2 Tukey HSD comparisons of growth rates. Download Table S2, XLSX file, 0.01 MB. Copyright © 2022 Sørum et al. 2022 Sørum et al. https://creativecommons.org/licenses/by/4.0/ This content is distributed under the terms of the Creative Commons Attribution 4.0 International license.

### Patterns of parallel evolution suggest compensatory mutations.

To identify the genetic basis for the observed changes in collateral effects as well as potential compensatory mutations, we subjected one fitness representative isolate from each of the evolved populations to whole-genome sequencing (*n* = 30; 1 clone from each CIP-R_evolved_ and WT_evolved_ population). After evolution, all CIP-R_evolved_ strains displayed putative compensatory mutations as indicated by mutations in genes modulating antimicrobial resistance, by parallel evolution, or both (Table 1). Parallel evolution is in general regarded as strong evidence for compensatory mutations (10, 21, 22).

We observed parallel evolution for two separate nonsynonymous mutations in *fyuA* (F87L popB; Q507P pop C) as well as an identical nonsynonymous *rpoS* (T298I) mutation in two out of three K56-2 CIP-R_evolved_ populations. FyuA is a TonB-dependent yersinabactin siderophore receptor associated with uropathogenic E. coli (23, 24). A mutation in *fyuA* (Q516P) was also identified in K56-70 CIP-R_evolved_. RpoS is a sigma factor subunit of the E. coli RNA polymerase and acts as regulator in several cellular mechanisms (25, 26). The RpoS interaction with the SOS response has also been linked to the maintenance of genome stability, to protection from genotoxic stressors, to altered susceptibility toward ciprofloxacin (27, 28), and as a target for compensatory mutation in fluoroquinolone-resistant Shigella sonnei (29). It has been shown previously that *gyrA* mutations reduce DNA supercoiling and cause upregulation of RpoS in Salmonella enterica (30). Moreover, different *gyrA* mutations have also been shown to reduce DNA supercoiling in E. coli to a variable degree (31), which in turn has been associated with regulation of TonB gene expression (32, 33).

We cannot completely rule out that mutations in *rpoS* and *fyuA* are adaptations to the experimental conditions. Mutations in *rpoS* have been shown to provide benefits in stationary phase (34), and we found a *rpoS* frameshift mutation in one of the evolved WT populations as well (Table S1). This population also contained a mutation in *mutS* and the high number of mutations indicate a mutator phenotype, and we interpretated these data with caution.

Based on the parallel evolution of nonsynonymous mutations, the absence of these exact mutations in WT_evolved_ populations taken together with the existing literature suggest that RpoS and potentially FyuA as a TonB-dependent receptor could function as targets for compensatory mutations.

In addition to the drug target mutation *gyrA* (S83L) in K56-41 CIP-R, K56-50 CIP-R, K56-70 CIP-R, and K56-78 CIP-R, all strains displayed mutations in genes related to drug efflux (4). Following experimental evolution, these strains also acquired additional mutations hypothesized to restore efflux activity to WT levels. K56-41 CIP-R initially had a 9-bp deletion in *rpoC*, and mutations in this gene have been associated with increased activity of the MdtK efflux pump (35). Following experimental evolution, we identified additional mutations in *rpoC* as well as in *rpoB*, which are also known to affect the MdtK efflux pump (35). These results indicate that the MdtK efflux system is the primary target for compensatory mutations in this strain background. In K56-50 CIP-R_evolved_, we identified a conversion of the initial RpoC S86F mutation into a F86C mutation in two of the three evolved populations (B and C), which likely restored WT RpoC activity due to the more similar physiochemical properties of cysteine and serine (WT). Population C also had an additional mutation in *rpoB* (V857E). K56-70 CIP-R displayed an initial mutation in RpoB (I668F) and a deletion in *marR*. All the evolved K56-70 CIP-R_evolved_ populations displayed amino acid changes in the same location as the initial amino acid change in RpoB (F668S, F668V, and F668L) indicating that the initial RpoB (I668F) mutation is a highly selective target for compensatory reversion mutations. Strain K56-78 CIP-R displayed mutations affecting both MdtK and the AcrAB-TolC efflux pumps. After experimental evolution, K56-78 CIP-R_evolved_ did not fully restore the cost of resistance, and parallel evolution was observed only in *dksA* in two of the evolved populations. DksA has been associated previously with ciprofloxacin tolerance functioning as a regulator of RNA polymerase in Yersinia pseudotuberculosis (36) as well as part of the ppGpp regulation in E. coli (37). If and how these mutations affect efflux levels are currently unknown. In two of the evolved CIP-R populations (K56-2 CIP-R_evolved_ pop A and K56-50 CIP-R_evolved_ pop A) and in two of the evolved WT populations (K56-41_evolved_ pop B and K56-78_evolved_ pop C), we identified mutations in *mutS* or *mutL*. Although this finding suggests a potential adaptive benefit of increased mutation rates in our evolution assay, these populations were handled with caution in the downstream analysis due to their complex mutational landscapes (Table S1).

### The cost of ciprofloxacin resistance mutations is dependent on genetic background.

The cost of drug target mutations observed in our clinical isolate (K56-2 CIP-R) was markedly larger than that in several earlier studies using emblematic laboratory strains of E. coli (38, 39), although variation in the literature exists with reports of larger costs more similar to our results (40). To further investigate the cost of these mutations in a different genetic background, we inserted the three initial mutations found in K56-2 CIP-R (*gyrA* S83L, *gyrA* A119E, and *parC* G78D) into E. coli MG1655. In the E. coli MG1655 background, our results were similar to earlier studies (38, 39) (w = 0.95, standard deviation = 0.031, *n* = 3). No other mutations were observed in K56-2 CIP-R (4) that could explain the difference in fitness cost, and we concluded that the large fitness effects observed here are due to strain specific differences, i.e., genetic background.

### Ancestral efflux phenotype was restored after evolution in the absence of antimicrobials.

**FIG 3:**
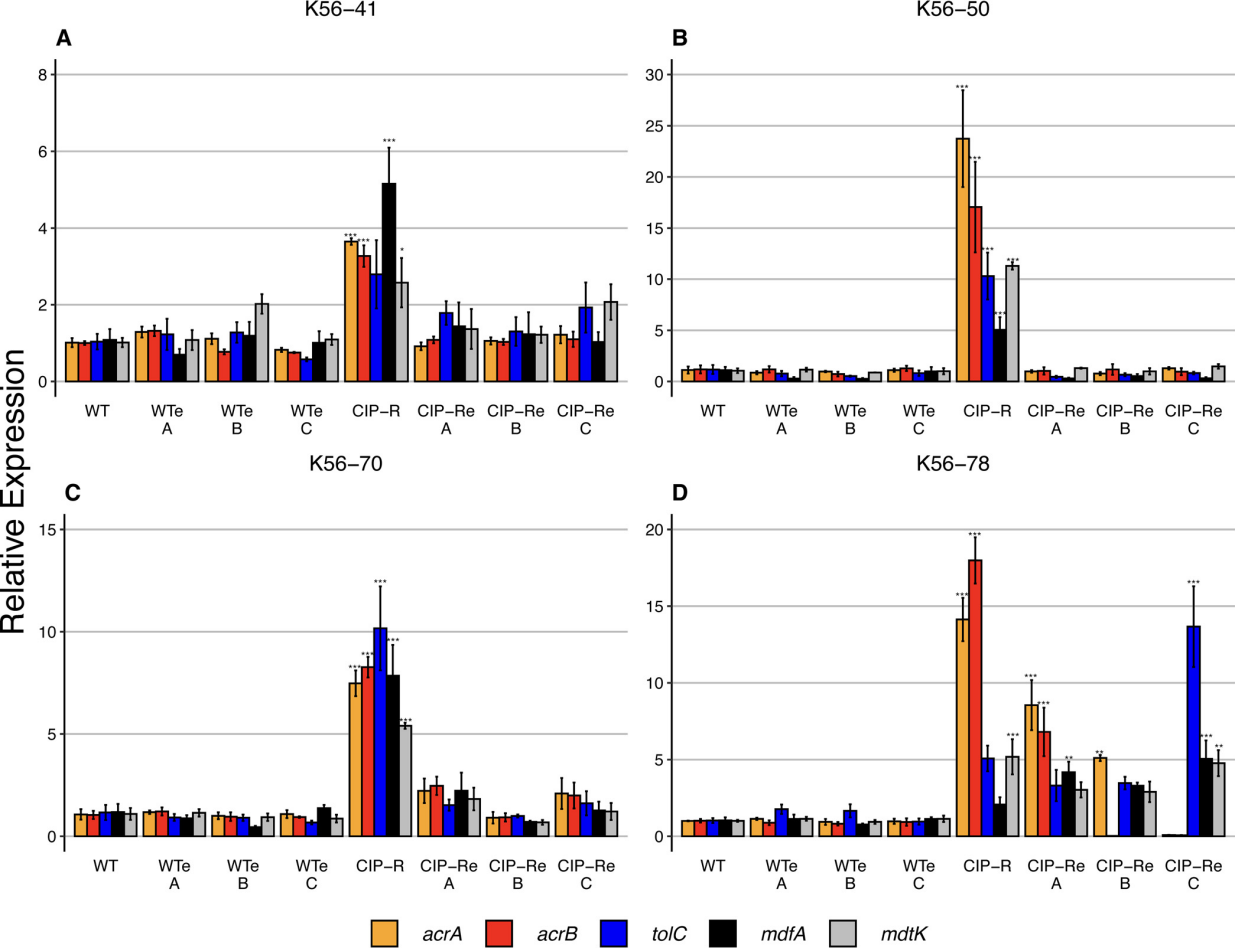
Relative transcription levels of identified efflux genes in the evolved WT (WT_e_), CIP-R mutants, and evolved CIP-R (CIP-R_e_) compared with their respective corresponding ancestral WT using reverse transcriptase quantitative PCR. A WT-WT comparison (far left on the *x* axis) was included as a control for each strain background. (A, B, and C) The different evolved populations for each strain. Increased efflux expression in CIP-R strains were restored to WT levels following evolution in all strains but K56-78. Columns represent the average value, and whiskers denote standard error; significance (***, *P* = 0.0001; **, *P* = 0.001; *, *P* = 0.05) from WT expression was adjusted for multiple comparisons with Dunnett’s test, *n* = 3.

To verify that initial ciprofloxacin resistance was due to the predicted increased efflux before experimental evolution, we functionally characterized the four strains with combinations of *gyrA* and efflux mutations (4). The expression of five structural efflux pump genes from efflux pumps (AcrAB-TolC [41], MdtK (NorE) [35], and MdfA [42]) with ciprofloxacin as one of their substrates was assessed by reverse transcriptase quantitative PCR (RT-qPCR). All four strains displayed increased transcription of *mdtK* and at least two genes associated with the AcrAB-TolC efflux pump compared with that of the WT (Fig. 3). In addition, we observed an increased expression of *mdfA* in all strains except K56-78 CIP-R. We demonstrated recently that the majority of collateral responses in these strains were due to mutations in efflux regulatory genes and not in *gyrA* (4). We further hypothesized that the loss of collateral phenotypes following experimental evolution was due to restored efflux pump activity in the absence of antimicrobial selection. RT-qPCR on evolved strains verified significant reduction in the expression of various efflux systems across all tested strains, strongly suggesting that reduced efflux levels were responsible for the loss of collateral responses as well as mitigations of fitness costs. The incomplete restoration of efflux systems to WT K56-78 CIP-R also provides an explanation for the observed partial fitness mitigation compared with that of the other strains (Fig. 3). The functional assessment of efflux genes tested here suggests that restoration of the WT efflux phenotype acts as the substrate/driver for fitness compensatory evolution and as the primary source for the loss of collateral sensitivity and resistance networks. Many of the antimicrobials used in this study are known substrates of the efflux pumps investigated in this study (Table 2).

**TABLE 2:** Drugs or drug classes used in this study known to interact with identified and related efflux pumps

Efflux pump	Results by antimicrobial^*a*^														References
	AMX	MEC	CAZ	ETP	FOS	COL	TET	TGC	GEN	AZT	CHL	CIP	NIT	SXT	
AcrAB-TolC	◊	◊	◊	◊			**+**	◊		◊	**+**	**+**		**+**	41, 56, 57
MdtK	◊	◊	◊	◊	**+**						**+**	**+**		◊	35, 58
MdfA							**+**	◊	◊	◊	**+**	**+**		◊	42, 58
Other efflux pumps known to require AcrA and TolC															
AcrAD-TolC	◊	◊	◊	◊					**+**			◊			57 – 59
Other relevant efflux pumps known to require TolC															
AcrEF-TolC	◊	◊	◊	◊			**+**	◊		◊	**+**	**+**		◊	56–58, 60
MdtABC-TolC	◊	◊	◊	◊								◊			57, 59, 61, 62
MdtEF-TolC	◊	◊	◊	◊						◊		**+**			57, 63
EmrAB-TolC												◊			

a **+**, evidence of efflux; ◊, evidence of drug class efflux. Antimicrobials from left to right: amoxicillin (AMX), mecillinam (MEC), ceftazidime (CAZ), ertapenem (ETP), fosfomycin (FOS), colistin (COL), tetracycline (TET), tigecycline (TGC), gentamicin (GEN), azithromycin (AZT), chloramphenicol (CHL), ciprofloxacin (CIP), nitrofurantoin (NIT), and trimethoprim-sulfamethoxazole (SXT).

## DISCUSSION

The clinical use of collateral networks to reduce the emergence and spread of antimicrobial resistance is contingent on a robust identification and predictions of collateral responses informed by susceptibility testing as well as the rapid detection of resistance mechanisms (4). Here, we demonstrate evolutionary instability of collateral networks in ciprofloxacin-resistant clinical E. coli UTI strains following 300 generations of experimental evolution in the absence of antimicrobial selective pressures. Our data strongly suggest that the evolutionary instability is caused by compensatory mutations reducing the initial fitness costs of ciprofloxacin resistance. In a recent study by Barbosa et al., they investigated the evolutionary stability of collateral sensitivity from a different perspective, subjecting P. aeruginosa to sequential exposure of antimicrobial pairs for which reciprocal collateral sensitivity was demonstrated (3). They identified several cases of resensitization to the first antimicrobial in a switch protocol following resistance development toward the second drug. This bacterial response occurred more frequently than the development of multidrug resistance, demonstrating that reciprocal collateral sensitivity is promising from a treatment perspective and effectively restricts bacteria in a “double bind evolutionary trap” (3). Our study is highly relevant downstream from the results presented by Barbosa et al. and underscore the importance of considering temporal evolutionary dynamics when predicting collateral networks.

Efflux-mediated ciprofloxacin resistance, the predominant resistance factor observed in the clinical isolates tested here, is widespread in the clinical setting and has been described in several reports investigating collateral networks across multiple species (9, 15). The high prevalence of resistance caused by an altered efflux phenotype is likely due to the large mutational target and high selection pressure protocols used to select for resistant isolates for comparison with selective ancestors. Although efflux is of clinical relevance in E. coli, it is not as prevalent as drug target mutations (43–46). However, here, we also identified instability of collateral effects in a mutant containing only drug target mutations, suggesting a generality of our findings with respect to ciprofloxacin-resistant E. coli. This result suggests that, at least for newly acquired resistance determinants, collateral networks should be treated as temporally unstable. The observation that fitness costs of resistance is a principal contributor of collateral networks (4) may not be universal and is likely linked to substantial initial fitness costs of newly acquired resistance determinants. This idea is supported by reports using E. coli MG1655 (2) and Streptococcus pneumoniae (47) where this link was not observed. Taken together with a recent report demonstrating that collateral networks are dependent on environmental factors (17), our data support that collateral responses measured for combinations of host strains and newly acquired resistance determinants across nonstandardized growth conditions serve as poor references for accurate predictions. To that end, the future clinical application of collateral responses requires careful considerations of evolutionary stability and the impact of biotic and abiotic factors on quantification and predictions of collateral effects as well as pharmacodynamics and pharmacokinetics of drug-bacterium combinations.

## MATERIALS AND METHODS

### Bacterial strains and strain constructions.

In this study, we assessed five clinical isolates of urinary tract infection E. coli from the ECO-SENS collection (48) that were selected previously for resistance *in vitro* (Table 1) by plating them onto increasing concentrations of ciprofloxacin (4). The five clinical isolates were selected based on their variation in ciprofloxacin resistance mutations providing a larger mutational landscape to study compensatory mutations. E. coli ATCC 25922 was used as a control strain in all IC_90_ assays. E. coli MG1655 was used as the recipient for *gyrA* (S83L; A119E) and *parC* (G78D) drug target mutations following the pORTMAGE protocol (49, 50) using the pORTMAGE-2 plasmid as described in reference 50. Oligonucleotides introducing the three different mutations used in the pORTMAGE cycling protocol was designed using MODEST (51). See Table S3 and S4 in the supplemental material for a complete list of bacterial strains and primers used in this study.

10.1128/mbio.00441-22.3 TABLE S3 Full list of bacterial strains used in this study. Download Table S3, XLSX file, 0.01 MB. Copyright © 2022 Sørum et al. 2022 Sørum et al. https://creativecommons.org/licenses/by/4.0/ This content is distributed under the terms of the Creative Commons Attribution 4.0 International license.

10.1128/mbio.00441-22.4 TABLE S4 Full list of primers and oligonucleotides used in this study. Download Table S4, XLSX file, 0.01 MB. Copyright © 2022 Sørum et al. 2022 Sørum et al. https://creativecommons.org/licenses/by/4.0/ This content is distributed under the terms of the Creative Commons Attribution 4.0 International license.

### Evolution in the absence of antimicrobials.

From five ciprofloxacin-resistant (CIP-R) mutants (4), three parallel populations (A, B, and C) and their corresponding WTs resulting in a total of 30 populations were evolved for 300 generations in the absence of antimicrobial selective pressure using a 1:100 serial transfer assay regime resulting in approximately 6.64 generations per transfer. Utilizing 96-well deep well plates, 10 μL from stationary-phase cultures was transferred into 990 μL fresh Muller-Hinton II broth (MHB; Becton, Dickinson and Company [BD], Franklin Lakes, NJ) every 24 h and incubated at 37°C shaking at 500 rpm. Evolving populations were grown in a checkerboard pattern with uninoculated growth medium to control for cross-contamination. After evolution, we identified a fitness representative colony from all evolved populations by testing three different isolates and included a population control. The isolate with the average growth rate was selected for each evolved populations and used for all further analysis.

### Susceptibility networks.

Susceptibility networks toward 15 antimicrobials (see Fig. 1) were obtained by assessing change in susceptibility testing using a broth microdilution assay (IC_90_ determination) (2, 4) before and after evolution. Overnight cultures of bacteria were suspended in sterile saline to make a 0.5 McFarland standard and diluted 1:1,000 in MHB. A total of 100 μL was transferred into 96-well microtiter plates containing a serial dilution of 1 of the 15 antibiotics following 1.5-fold dilution steps, and 200 μL was the final volume. The plates were incubated shaking at 37°C, 700 rpm, for 18 h. Optical density at 600 nm (OD_600_) was measured in a Versamax plate reader (Molecular Devices Corporation, CA). IC_90_ was calculated as OD_600_ antibiotic concentration × OD_600_ positive control^−1^ (2). Results were based on at least three biological replicates for each population that were performed on different days, and all plates included the E. coli ATCC 25922 test strain as a control. From the same raw data used for IC_90_ determination, we also obtained dose response curves (Fig. S2) where OD_600_ was normalized to percent growth at different antimicrobial concentrations. Curves were fitted to the data using a variable slope model forcing the curve to stay within the outer boundaries of 100% and 0% using Prism (v.9.1.1; GraphPad Software, San Diego, CA).

### Growth rates.

Growth curves of the evolved populations of WT_evolved_ and CIP-R_evolved_ were obtained by inoculating at least three biological replicates into 2 mL of MHB which was incubated at 37°C and 500 rpm for 24 h. The starting cultures were then diluted 1:100 resulting in a starting titer of ~2 × 10^7^ cells mL^−1^. From these dilutions, 250 μL was added to a 96-well microtiter plate in triplicates. The plate was incubated overnight in a Versamax plate reader (Molecular Devices Corporation, CA) at 37°C and shaking for 9.2 min between each read. Optical density (OD_600_) measurements were taken every 10 min, and growth rates (r) were estimated using GrowthRates v.2.1 (52). The reported relative growth rates (w) were obtained using the formula r_(CIP-Revolved)_ × r_(WTevolved)_^−1^. One-way analysis of variance (ANOVA) tests adjusted for multiple comparisons with Tukey honestly significant difference (HSD) tests were used to assess significant changes in growth rates. Significance was considered at a *P* value of ≤0.05.

### Whole-genome sequencing.

Genomic DNA was isolated from a single colony of a fitness representative biological replicate in the growth rate assay from each of the parallel evolved CIP-R and WT populations. The WT_evolved_ populations were sequenced to control for adaptations to growth medium and experimental conditions. We used GenElute for bacteria genomic DNA kit (Sigma-Aldrich, St. Louis, MO) following the gram-positive DNA extraction protocol to increase DNA isolation yield in our clinical strains. The purity of the samples was assessed using NanoDrop (Thermo Fisher), and DNA quantification was performed using a Qubit high-sensitivity DNA assay (Life Technologies). Sequencing was performed according to the Nextera XT DNA library prep kit (Illumina, San Diego, CA) at the Genomics Resource Centre Tromsø (UiT Arctic University of Norway). A downstream analysis of DNA sequences was performed according to Podnecky et al. 2018 (4) to allow for direct comparisons of the sequence data. In short, raw reads from the evolved WT_evolved_ and CIP-R_evolved_ were aligned to previously annotated (RAST 2.0 for E. coli [53]) sequences of the WT strains utilizing standard settings in SeqMan NGen (DNASTAR, Madison, WI). Minimum values of reported single nucleotide polymorphisms (SNPs) were 10× coverage depth and 90% variant base calls. Reported SNPs and indels were inspected manually.

### Relative gene expression.

To assess relative transcription levels, reverse transcriptase quantitative PCR (RT-qPCR) was performed. Samples were grown in MHB overnight at 37°C with shaking (200 rpm). A total of 500 μL of mid-log-phase (OD_600_ of 0.45 to 0.7, strain specific) cells were stabilized using RNA protect bacteria reagent (Qiagen, Hilden, Germany), and cell pellets were stored at −80°C. Total RNA was extracted using the RNeasy minikit (Qiagen). A total of 1 μg of RNA was treated using a DNA-free DNA removal kit (Invitrogen, Carlsbad, CA) with a 2× concentration of rDNase I, as recommended for rigorous DNase treatment. cDNA synthesis was performed on 4 μL of DNase-treated RNA using SuperScript III first-strand synthesis SuperMix for qRT-PCR (Invitrogen). The DNase treatment and cDNA synthesis protocols were upscaled as needed. RT-qPCR was performed using the PowerUp SYBR green master mix (Thermo Fisher) on a 7300 real-time PCR system and 7300 sequence detection system (SDS) relative quantification (RQ) study software (Applied Biosystems, Beverly Hills, CA). Melt curve analyses were used to rule out secondary products and primer dimer formation. Samples were tested in technical triplicate, and threshold cycle (*C_T_*) values were averaged for analysis. CysG was used for data normalization; primer sequences are listed in Table 3. Relative expression to the respective WT or CIP-R isolate was calculated using the ΔΔ*C_T_* method (54). Relative expression was assessed in at least biological triplicate; additional replicates were performed if the relative expression varied more than 4-fold between biological replicates and the outlier excluded, if applicable. Average relative expression and standard error were calculated, Dunnett’s test controlling for multiple comparisons was used to assess significant changes in gene expression. Significance was considered at a *P* value of ≤0.05.

**TABLE 3:** RT-qPCR primers used in this study

Gene	Primer sequence (5′–3′)		Reference
	Forward	Reverse	
*cysG*	TTGTCGGCGGTGGTGATGTC	ATGCGGTGAAACTGTGGAATAAACG	35
*acrA*	CTCTCAGGCAGCTTAGCCCTAA	TGCAGAGGTTCAGTTTTGACTGTT	64
*acrB*	GGTCGATTCCGTTCTCCGTTA	CTACCTGGAAGTAAACGTCATTGGT	64
*tolC*	AAGCCGAAAAACGCAACCT	CAGAGTCGGTAAGTGACCATC	64
*mdfA*	CATTGGCAGCGATCTCCTTT	TTATAGTCACGACCGACTTCTTTCA	64
*mdtK/norE*	CTGGCGGCAGCGGTAA	TGCCATACAGACACCCACCATA	64

### Data analysis and software.

Statistical analyses were performed using R (v.4.0.3) (55) and Prism (v.9.1.1; GraphPad Software, San Diego, CA). Growth rates were obtained from GrowthRates v.2.1 (52).
