# Supplementary material for: Evolutionary Instability of Collateral Susceptibility Networks in Ciprofloxacin-Resistant Clinical Escherichia coli Strains
Source: mBio. 2022 Jul 7;13(4):e00441-22. doi: 10.1128/mbio.00441-22 (PMC9426462; doi:10.1128/mbio.00441-22)
Supplement: FIG S2 [file mbio.00441-22-s0006.pdf]

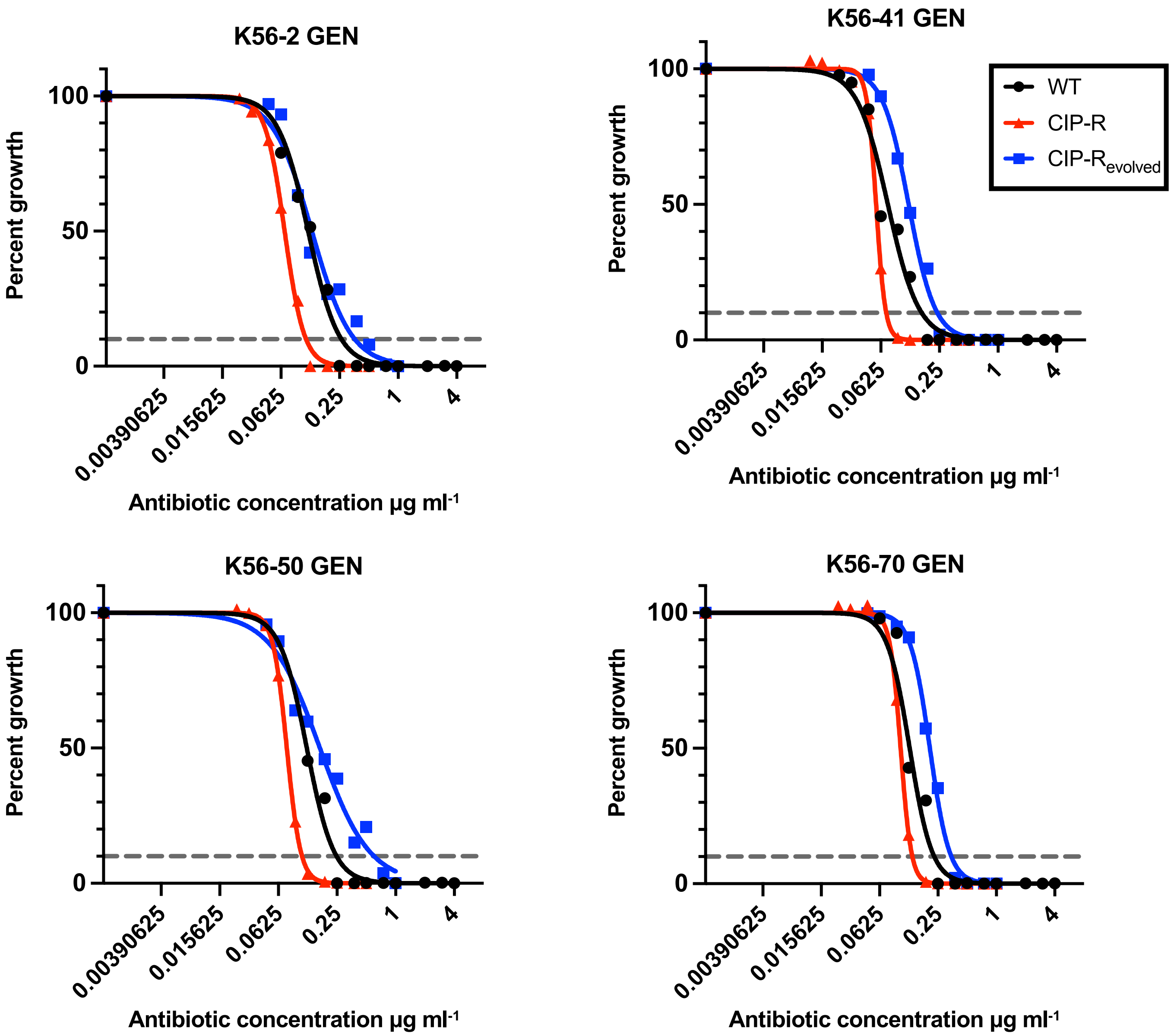

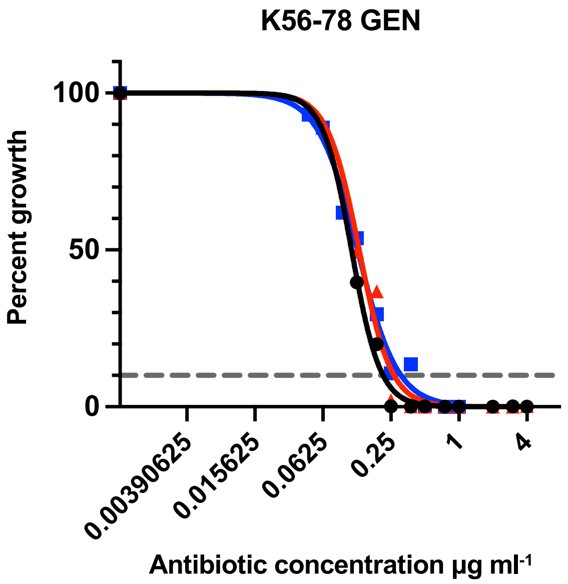

Supplementary figure 2 – Gentamicin:
Dose response curves denoting percent growth at given antibiotic concentrations for Ancestral WT (black circles), Ancestral ciprofloxacin resitant (red triangles) and Evolved ciprofloxacin restant (blue squares). Each strain background are separated into different graphs. Grey dashed line denotes the IC_{90}. Shown values are averages and CIP-R$_{evolved}$ is based on the average of the three evolved populations.

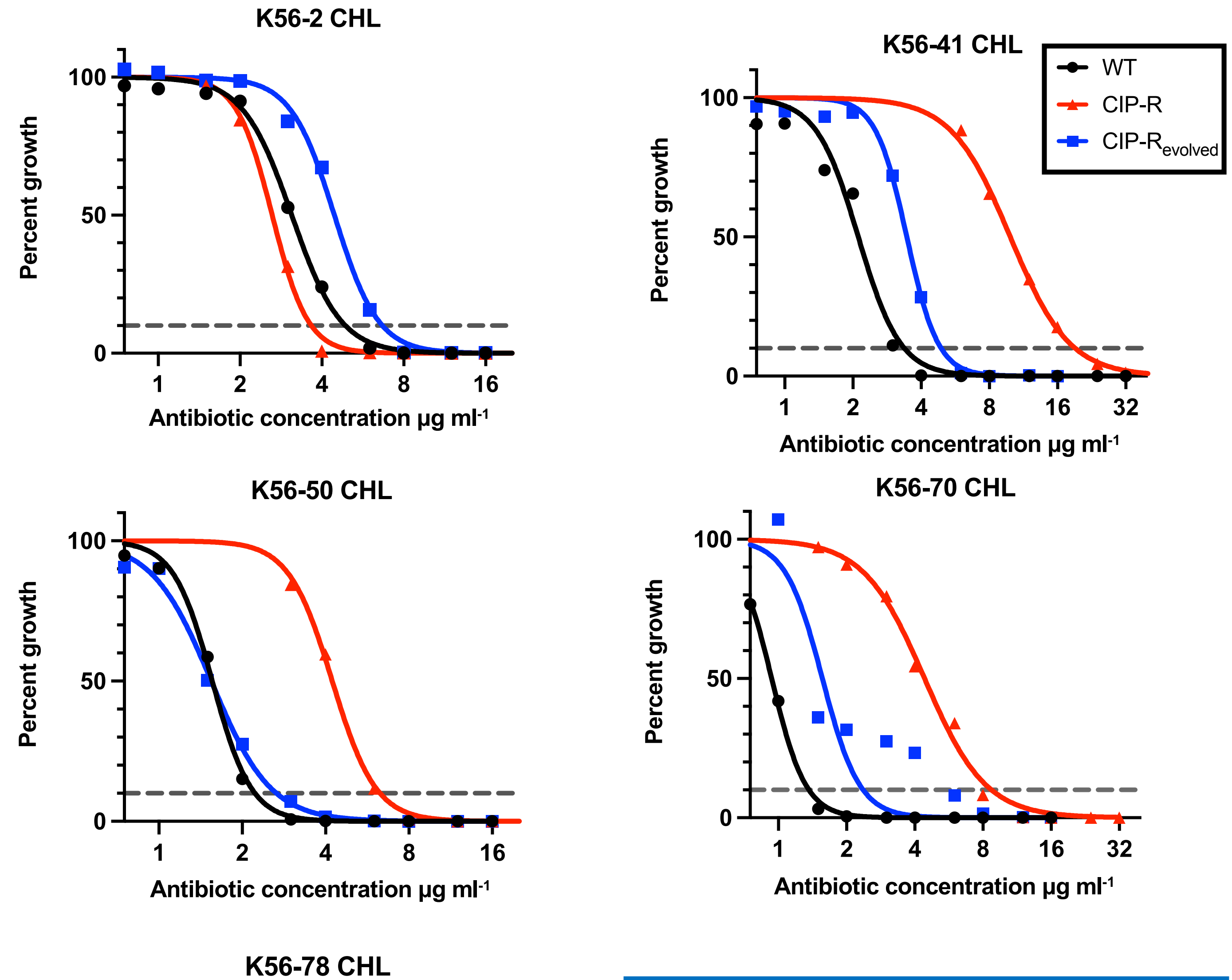

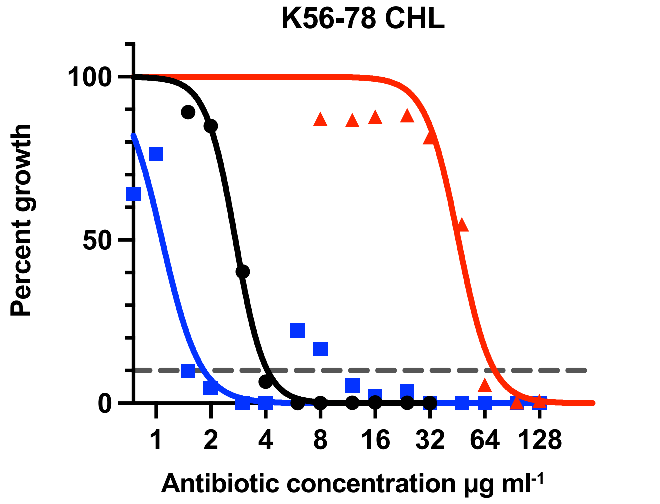

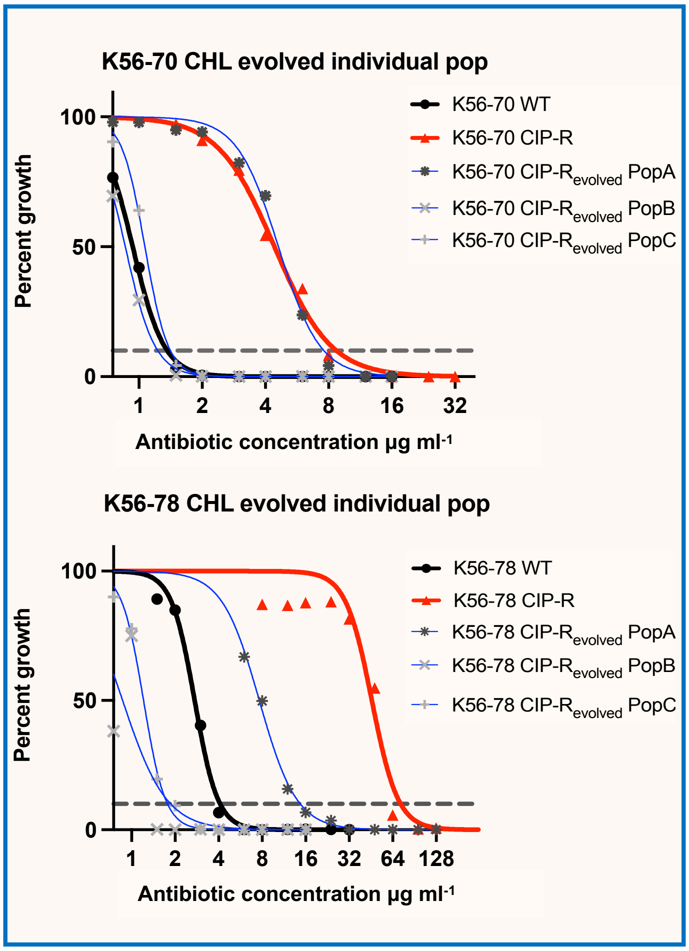

Supplementary figure 2 – Chloramphenicol:
Dose response curves denoting percent growth at given antibiotic concentrations for Ancestral WT (black circles), Ancestral ciprofloxacin resitant (red triangles) and Evolved ciprofloxacin restant (blue squares). Each strain background are separated into different graphs. Shown values are averages and CIP-$R_{evolved}$ is based on the average of the three evolved populations. Framed in the lower right corner the three individually evolved populations (grey symbols and blue lines) for strain K56-70 and K56-78 is shown separately due to large variation between the populations. Grey dashed line denotes the IC_{90}.

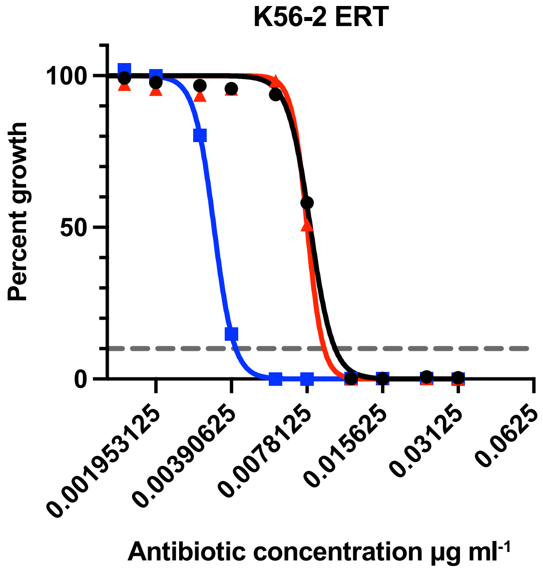

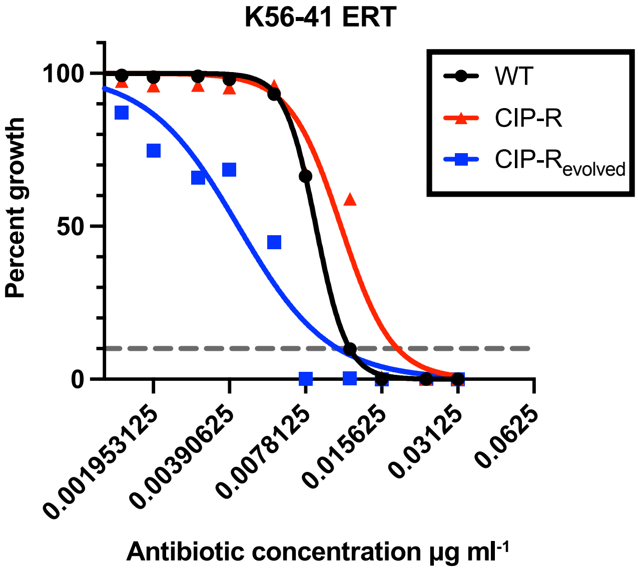

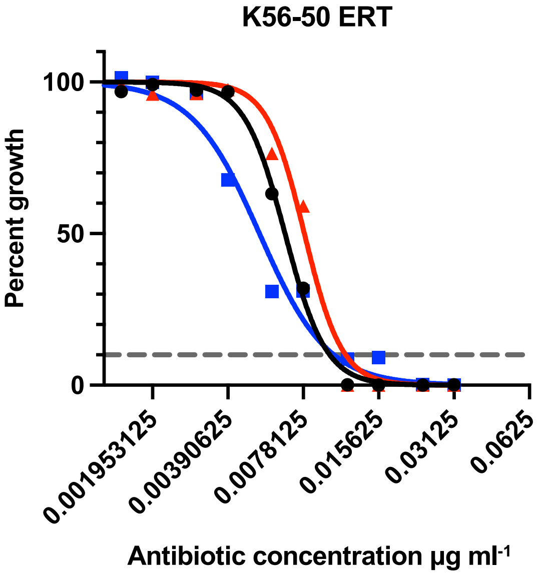

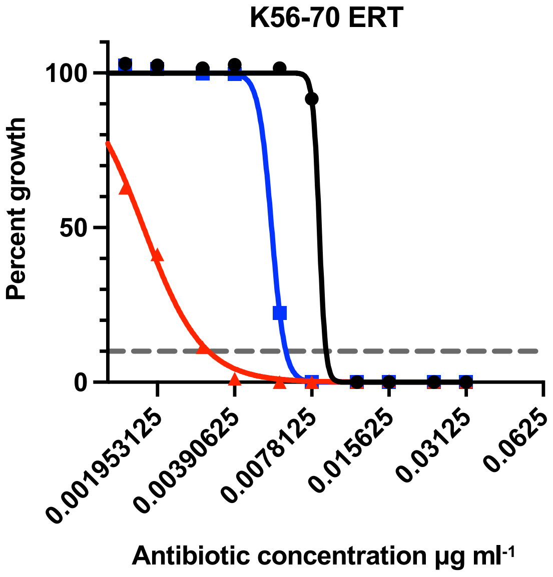

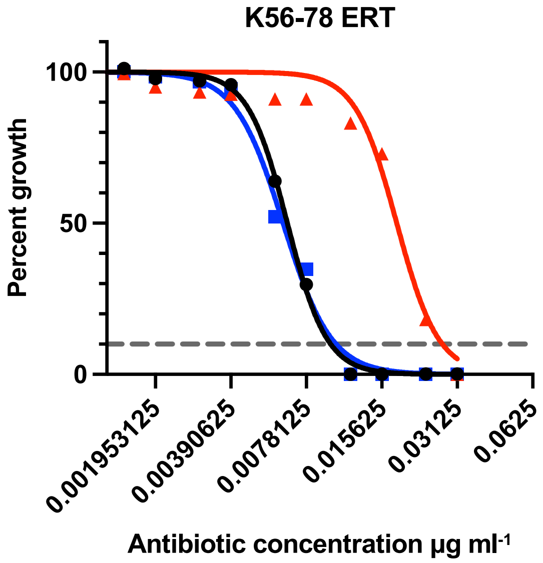

Supplementary figure 2 – Ertapenem:
Dose response curves denoting percent growth at given antibiotic concentrations for Ancestral WT (black circles), Ancestral ciprofloxacin resitant (red triangles) and Evolved ciprofloxacin restant (blue squares). Each strain background are separated into different graphs. Grey dashed line denotes the IC_{90}. Shown values are averages and CIP-$R_{evolved}$ is based on the average of the three evolved populations.

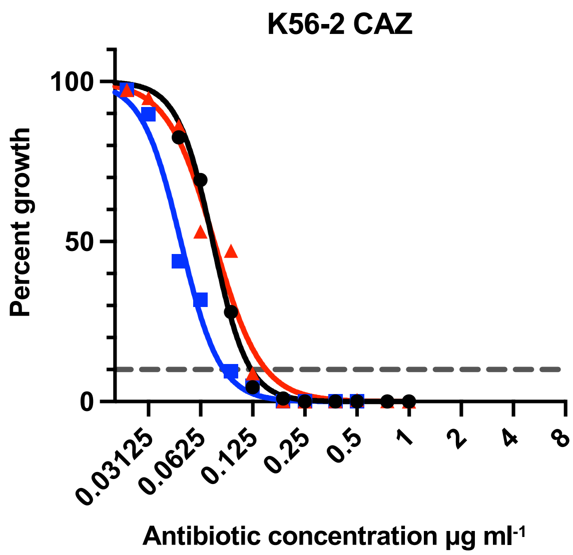

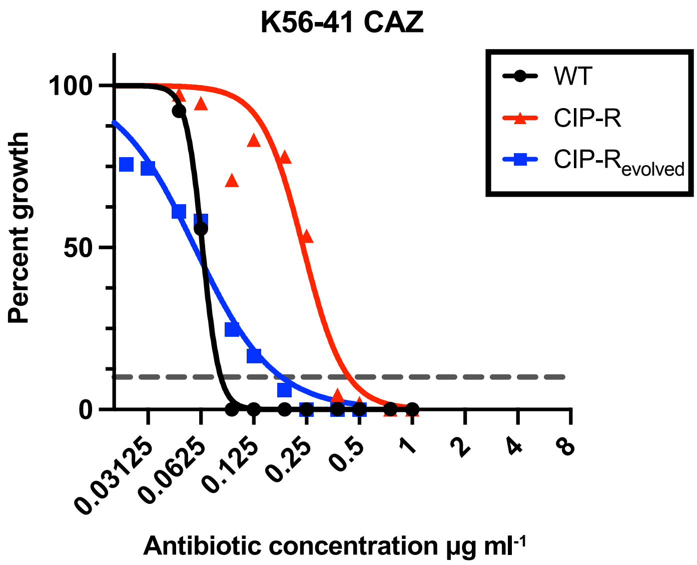

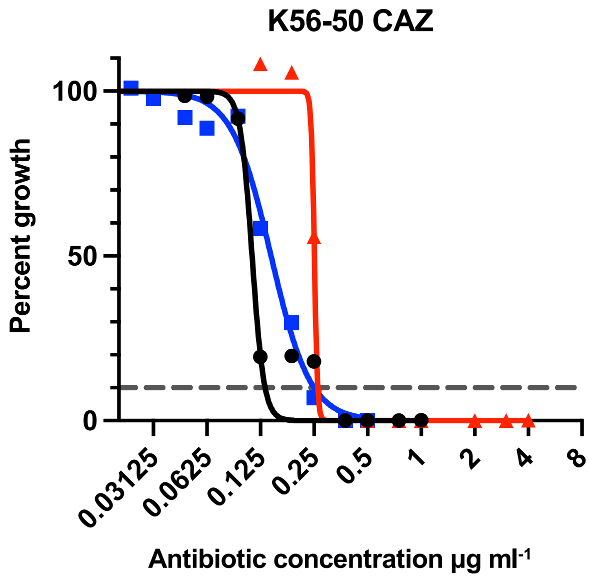

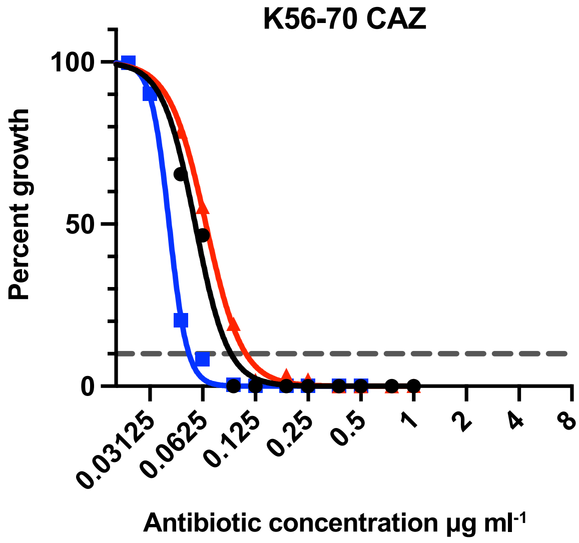

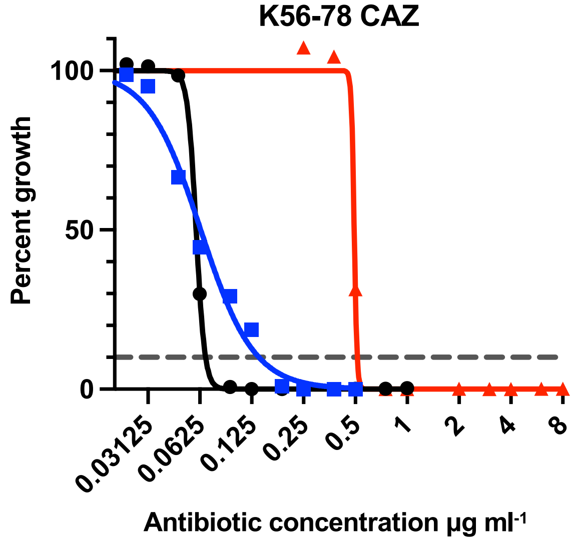

Supplementary figure 2 – Ceftazidime:
Dose response curves denoting percent growth at given antibiotic concentrations for Ancestral WT (black circles), Ancestral ciprofloxacin resitant (red triangles) and Evolved ciprofloxacin restant (blue squares). Each strain background are separated into different graphs. Grey dashed line denotes the IC_{90}. Shown values are averages and CIP-$R_{evolved}$ is based on the average of the three evolved populations.

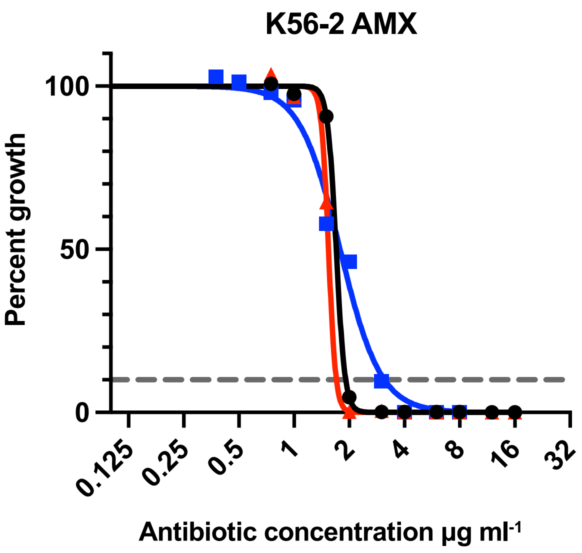

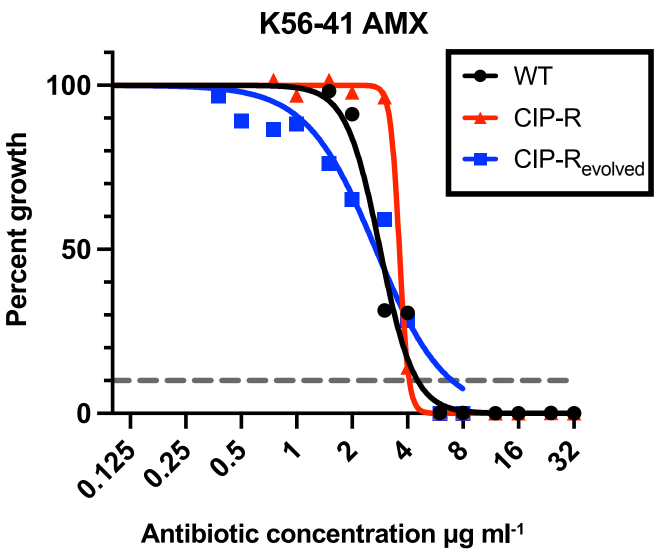

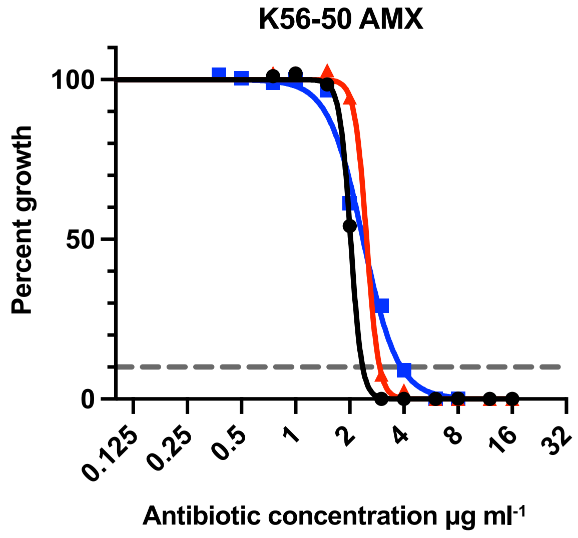

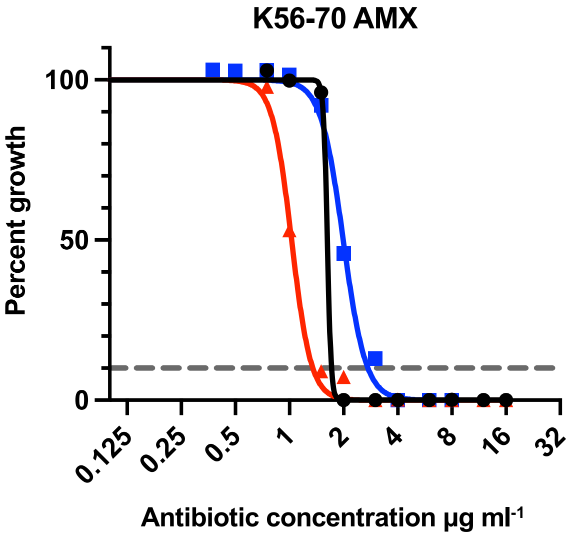

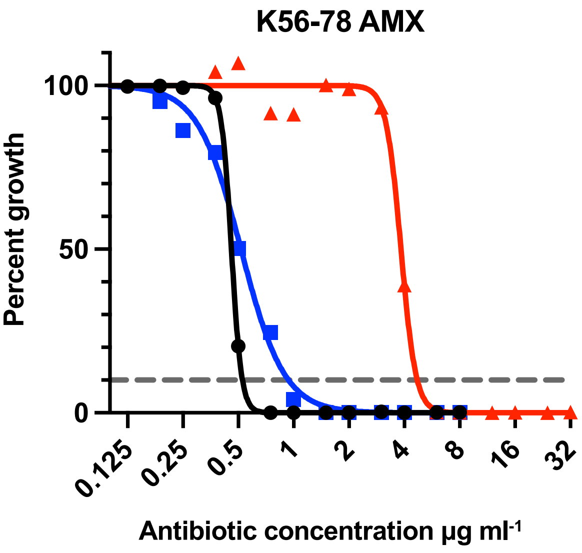

Supplementary figure 2 – Amoxicillin:
Dose response curves denoting percent growth at given antibiotic concentrations for Ancestral WT (black circles), Ancestral ciprofloxacin resitant (red triangles) and Evolved ciprofloxacin restant (blue squares). Each strain background are separated into different graphs. Grey dashed line denotes the IC_{90}. Shown values are averages and CIP-$R_{evolved}$ is based on the average of the three evolved populations.

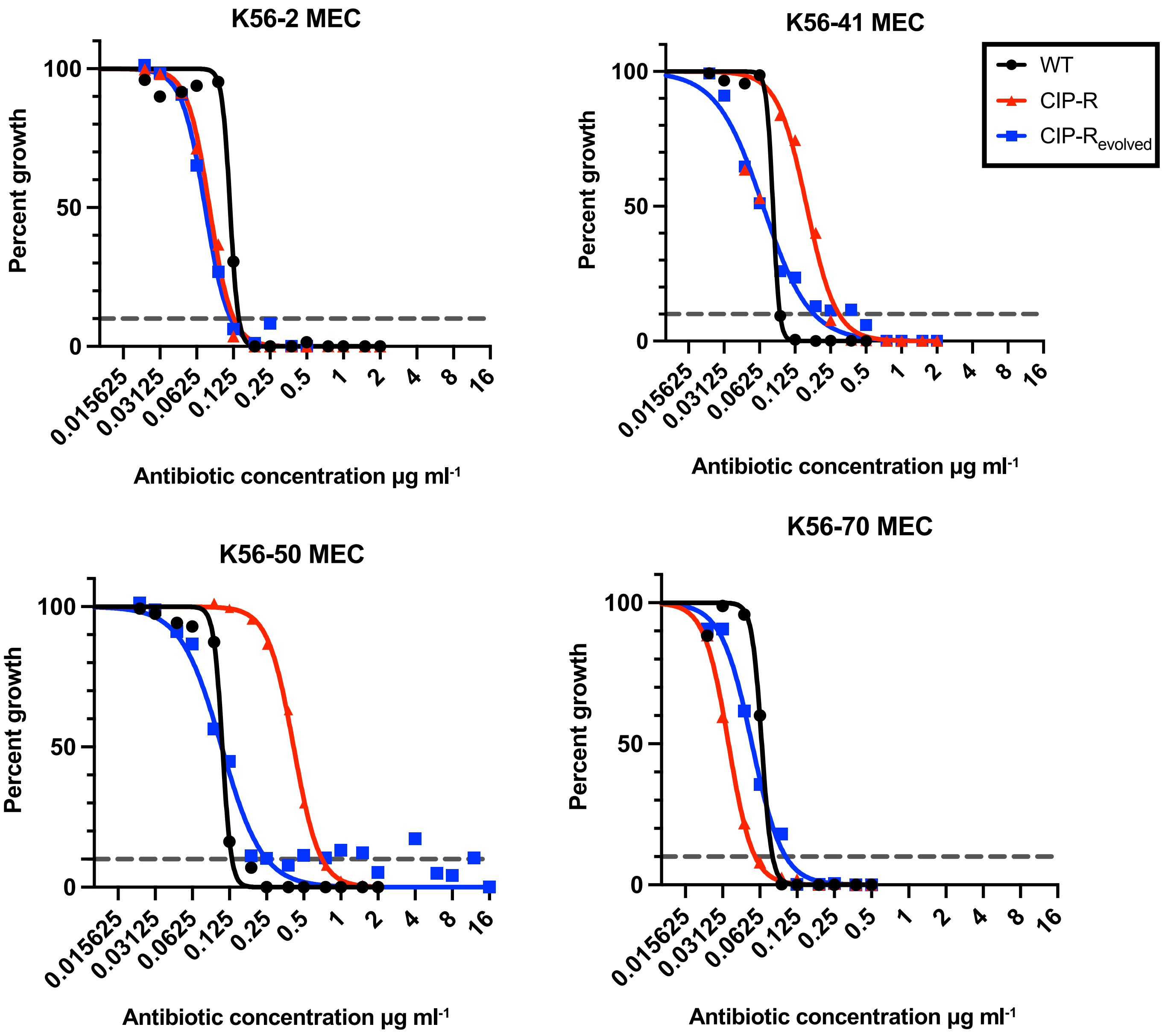

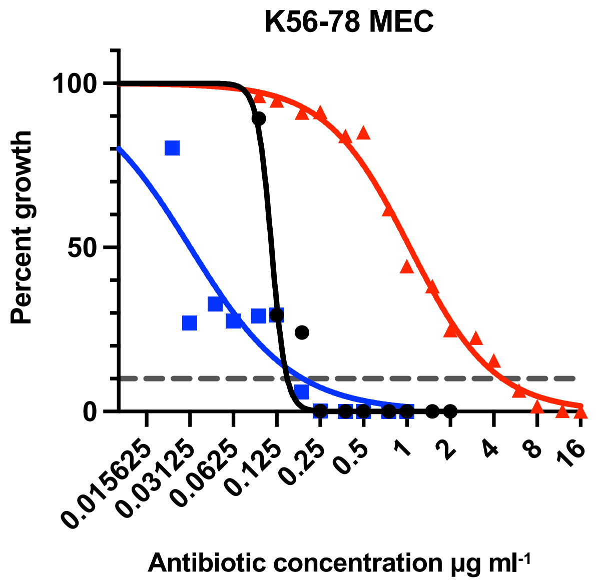

Supplementary figure 2 – Mecillinam:
Dose response curves denoting percent growth at given antibiotic concentrations for Ancestral WT (black circles), Ancestral ciprofloxacin resitant (red triangles) and Evolved ciprofloxacin restant (blue squares). Each strain background are separated into different graphs. Grey dashed line denotes the IC_{90}. Shown values are averages and CIP-R$_{evolved}$ is based on the average of the three evolved populations.

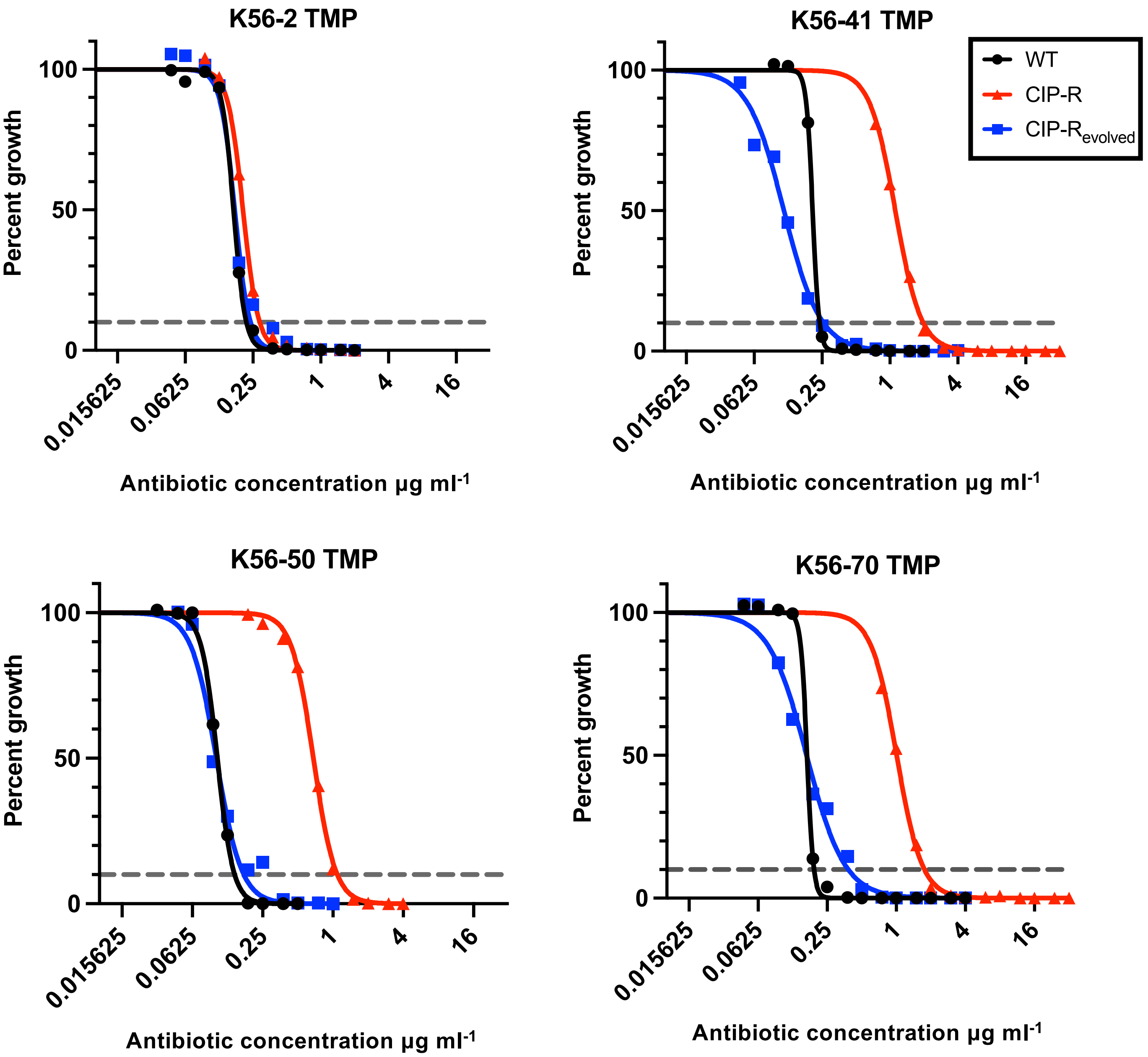

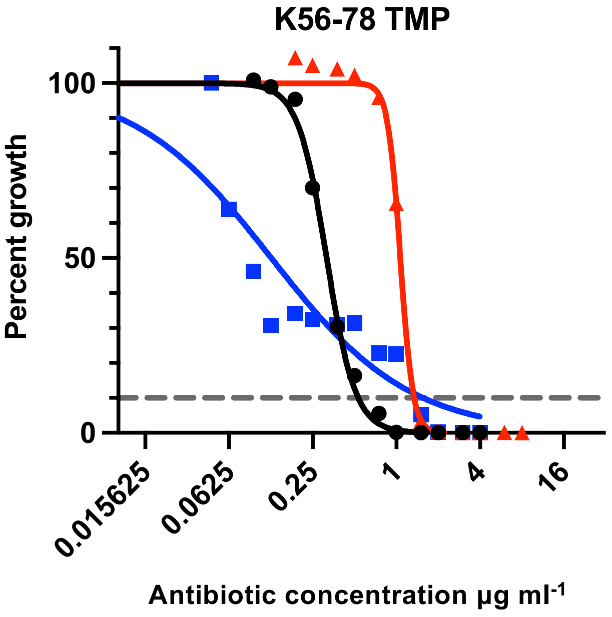

Supplementary figure 2 – Trimethoprim:
Dose response curves denoting percent growth at given antibiotic concentrations for Ancestral WT (black circles), Ancestral ciprofloxacin resitant (red triangles) and Evolved ciprofloxacin restant (blue squares). Each strain background are separated into different graphs. Grey dashed line denotes the IC_{90}. Shown values are averages and CIP-R$_{evolved}$ is based on the average of the three evolved populations.

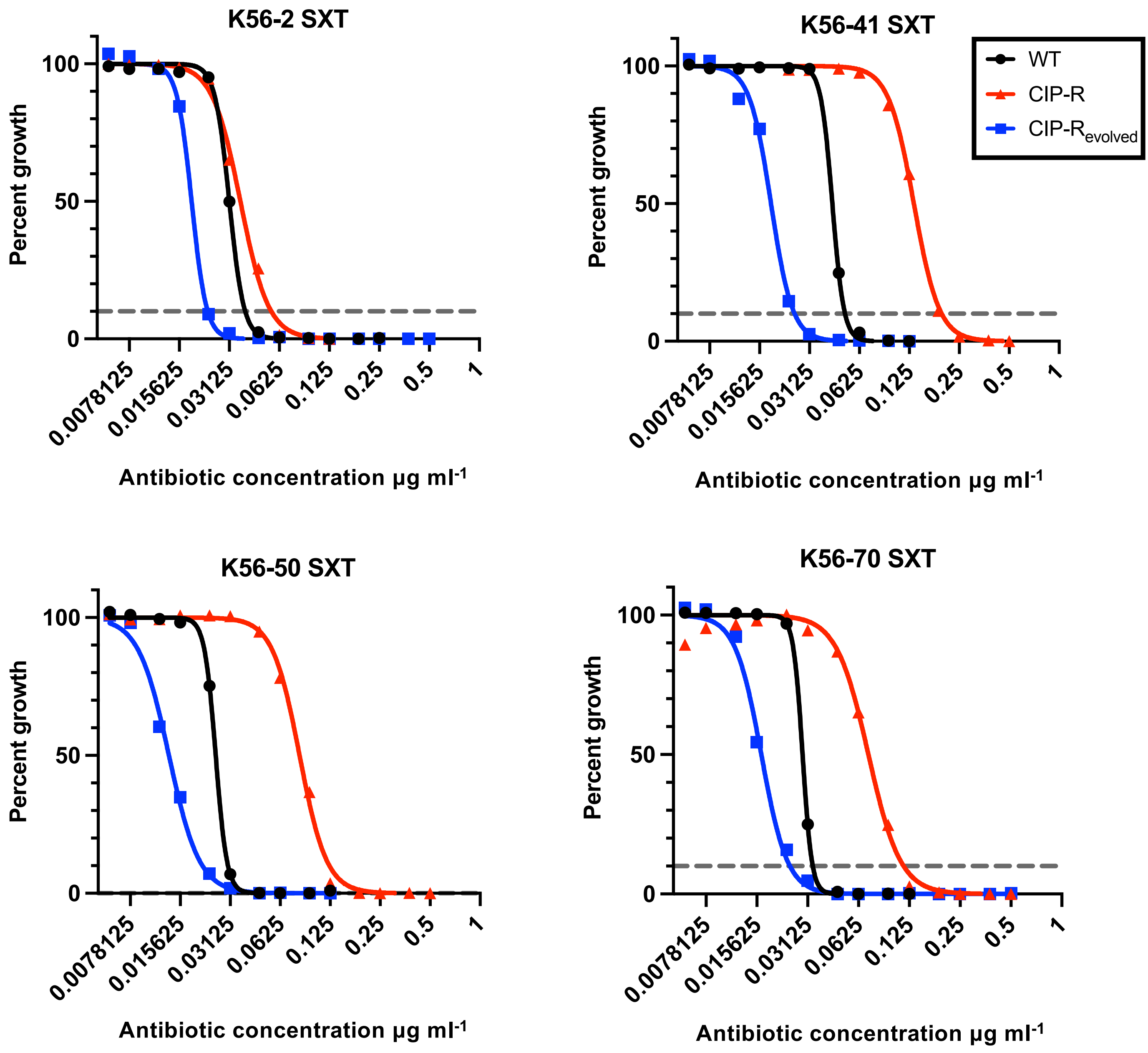

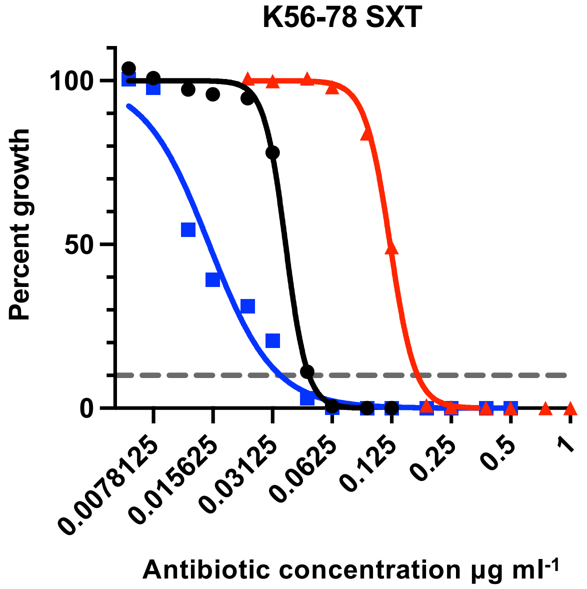

Supplementary figure 2 – Trimethoprim-Sulfametoxazole:

Dose response curves denoting percent growth at given antibiotic concentrations for Ancestral WT (black circles), Ancestral ciprofloxacin resitant (red triangles) and Evolved ciprofloxacin restant (blue squares). Each strain background are separated into different graphs. Grey dashed line denotes the IC_{90}. Shown values are averages and CIP-R$_{evolved}$ is based on the average of the three evolved populations.

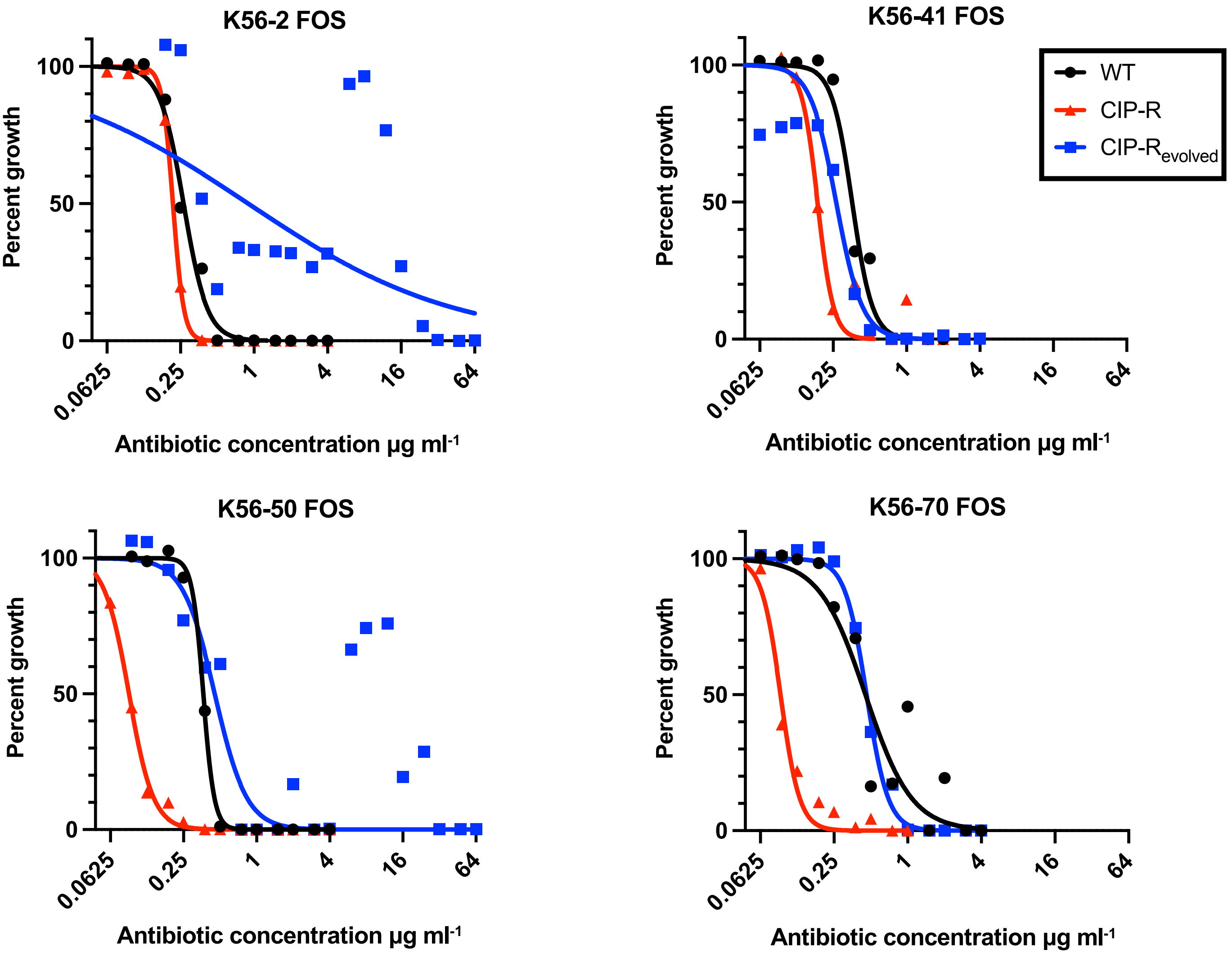

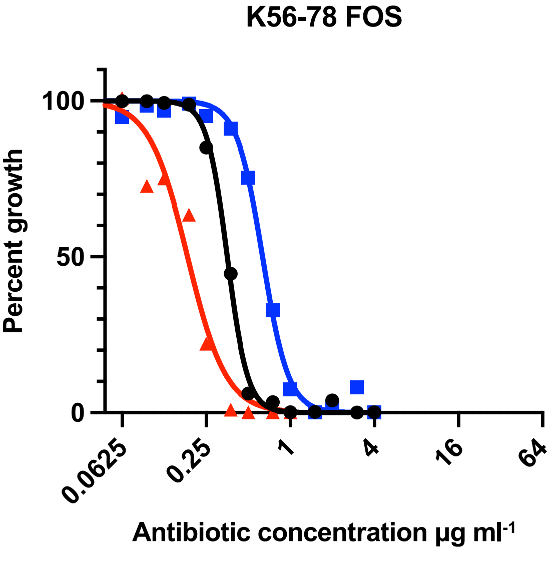

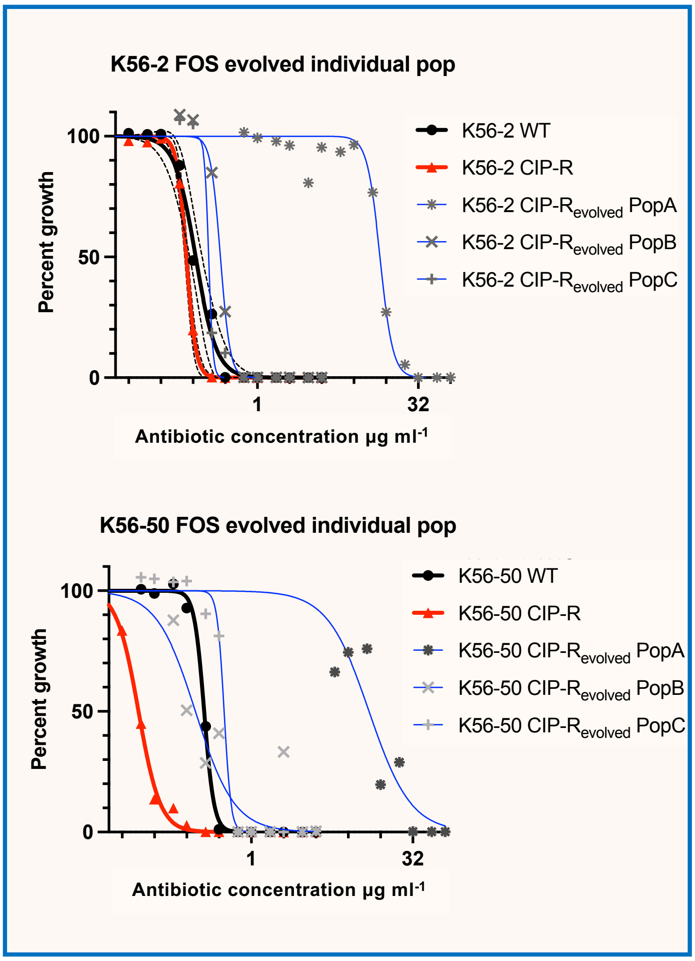

Supplementary figure 2 – Fosfomycin:
Dose response curves denoting percent growth at given antibiotic concentrations for Ancestral WT (black circles), Ancestral ciprofloxacin resitant (red triangles) and Evolved ciprofloxacin restant (blue squares). Each strain background are separated into different graphs. Shown values are averages and CIP-$R_{evolved}$ is based on the average of the three evolved populations. Framed in the lower right corner the three individually evolved populations (grey symbols and blue lines) for strain K56-2 and K56-50 is shown separately due to large variation between the populations. Grey dashed line denotes the IC_{90}.

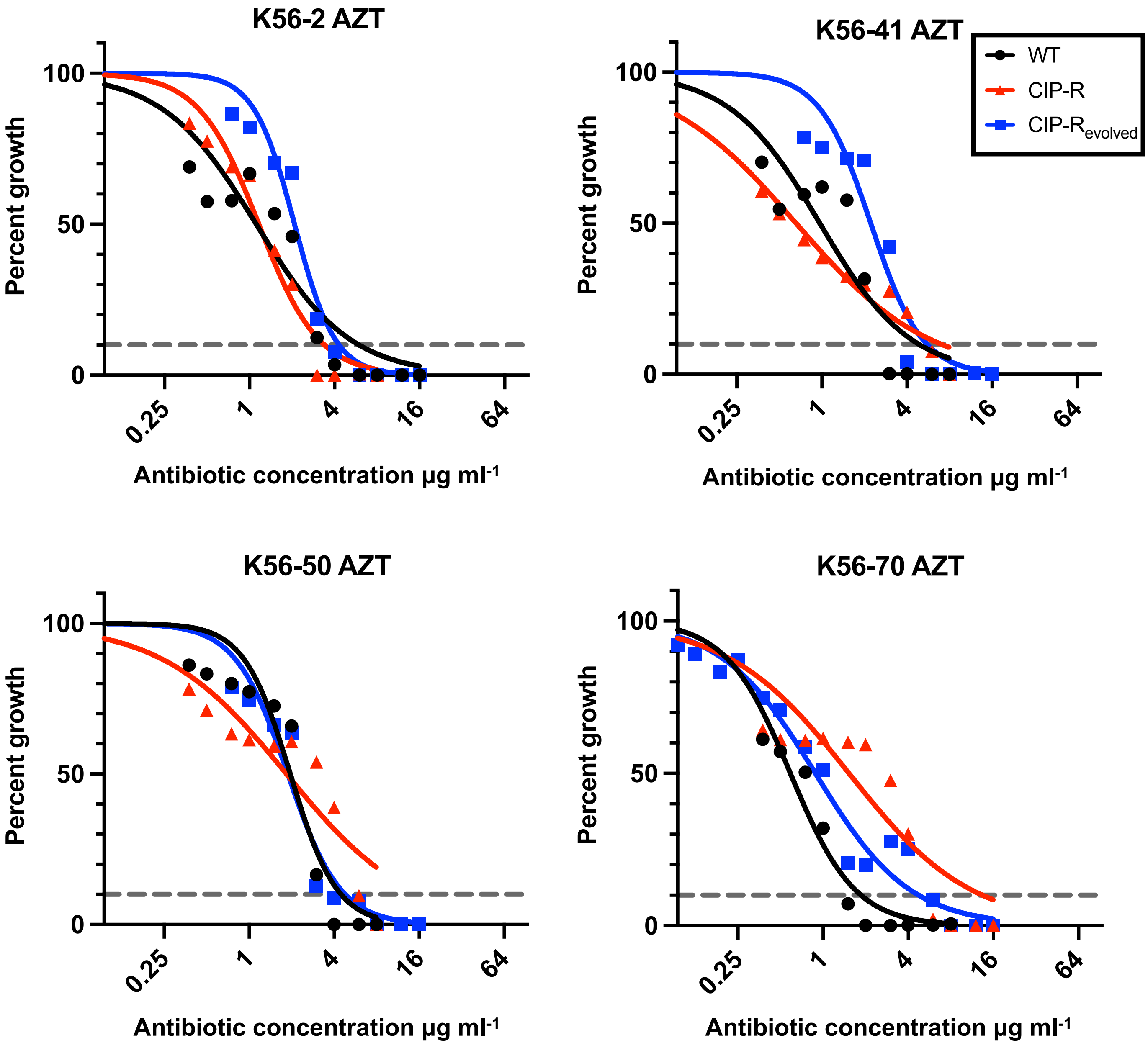

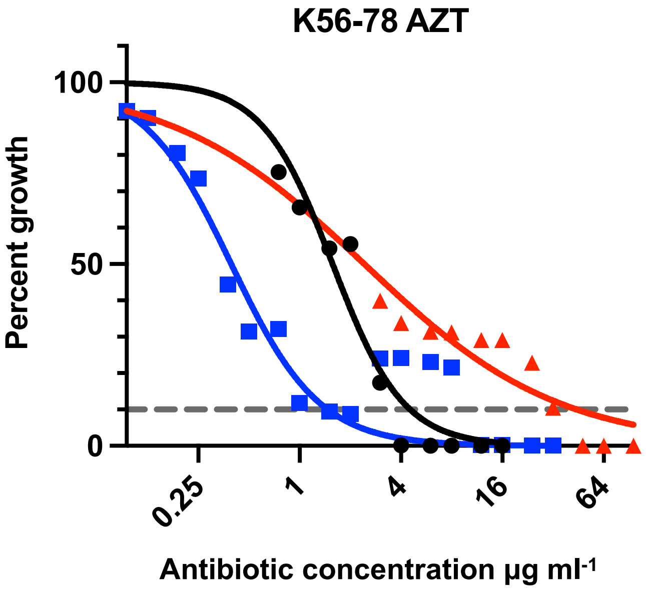

Supplementary figure 2 – Azithromycin:
Dose response curves denoting percent growth at given antibiotic concentrations are provided for Ancestral WT (black circles), Ancestral ciprofloxacin resitant (red triangles) and Evolved ciprofloxacin restant (blue squares). The different strain backgrounds are separated into different graphs. Grey dashed line denotes the IC_{90}. Shown values are averages and CIP-$R_{evolved}$ is based on the average of the three evolved populations.

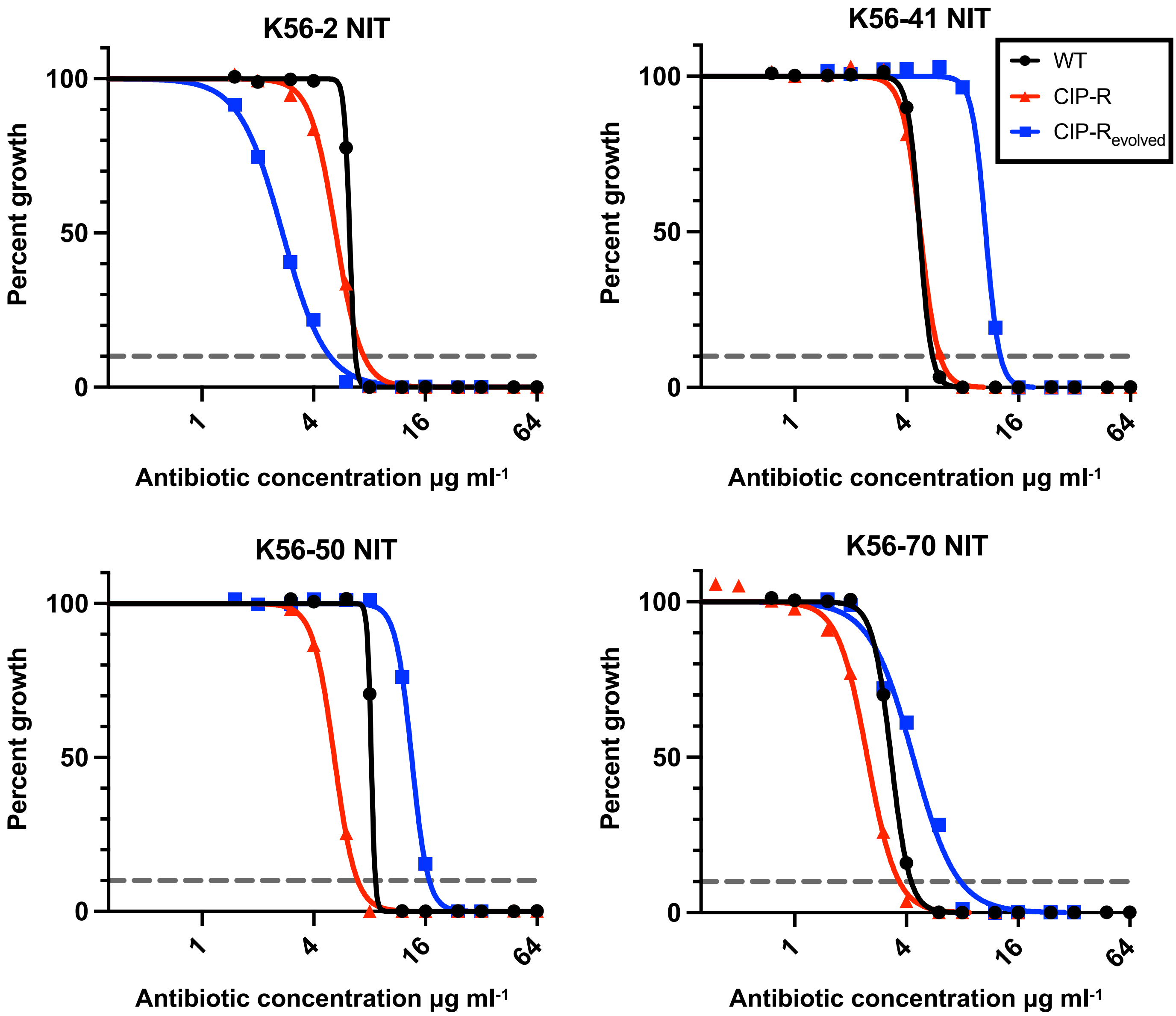

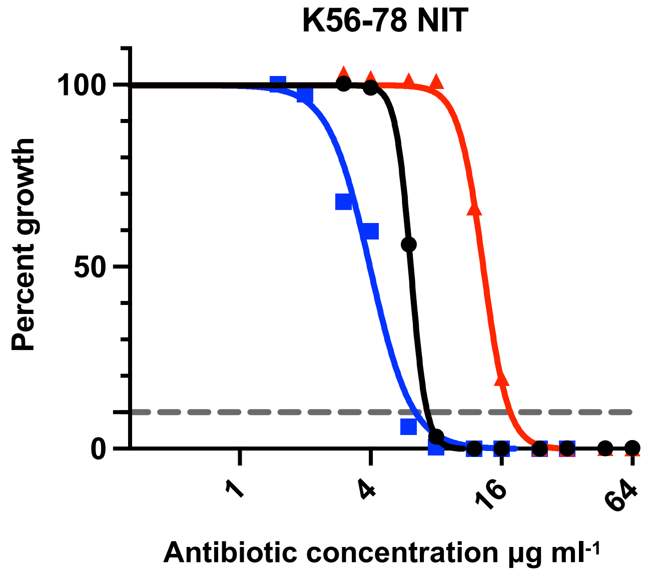

Supplementary figure 2 – Nitrofurantoin:
Dose response curves denoting percent growth at given antibiotic concentrations for Ancestral WT (black circles), Ancestral ciprofloxacin resitant (red triangles) and Evolved ciprofloxacin restant (blue squares). Each strain background are separated into different graphs. Grey dashed line denotes the IC_{90}. Shown values are averages and CIP-R$_{evolved}$ is based on the average of the three evolved populations.

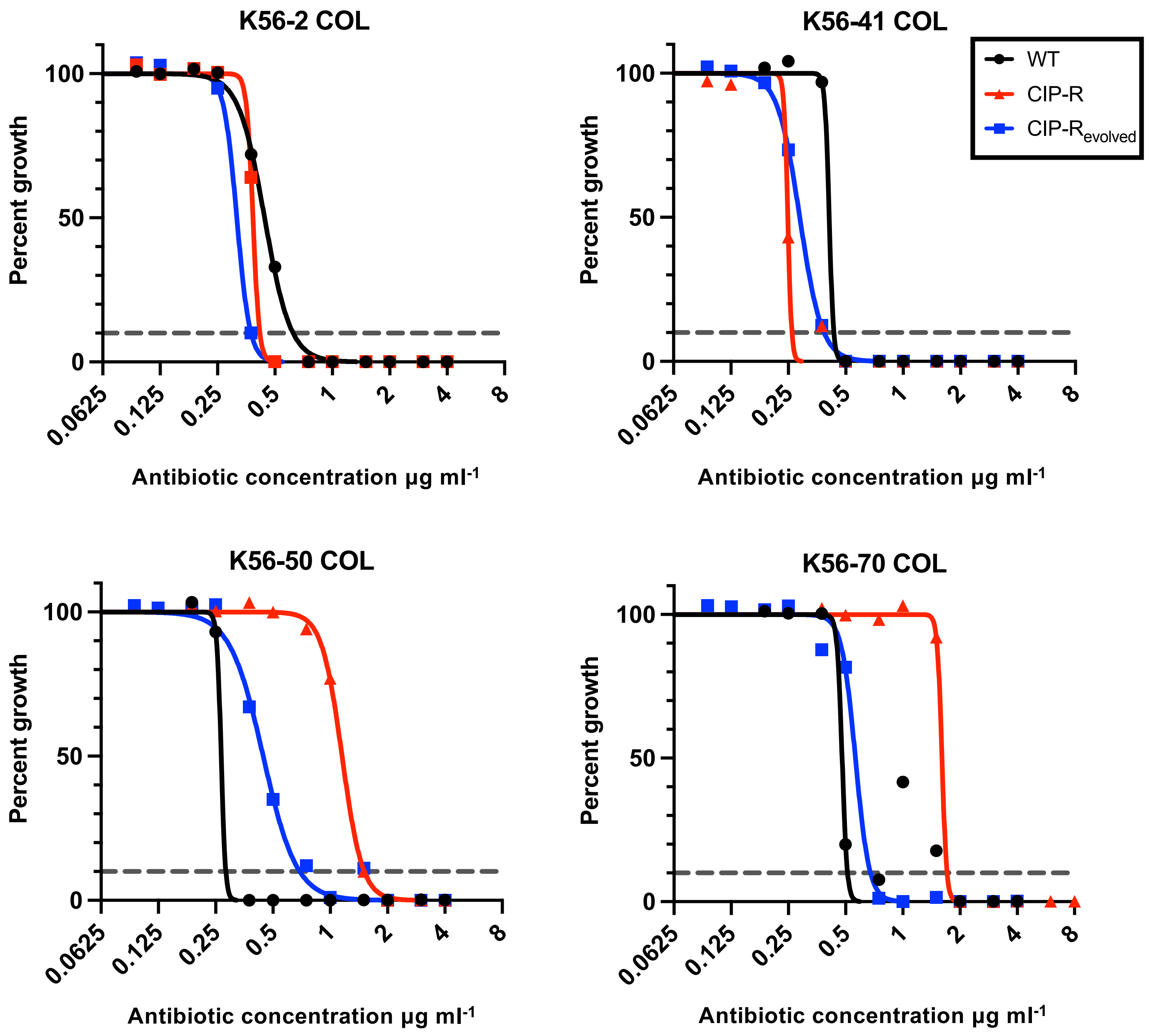

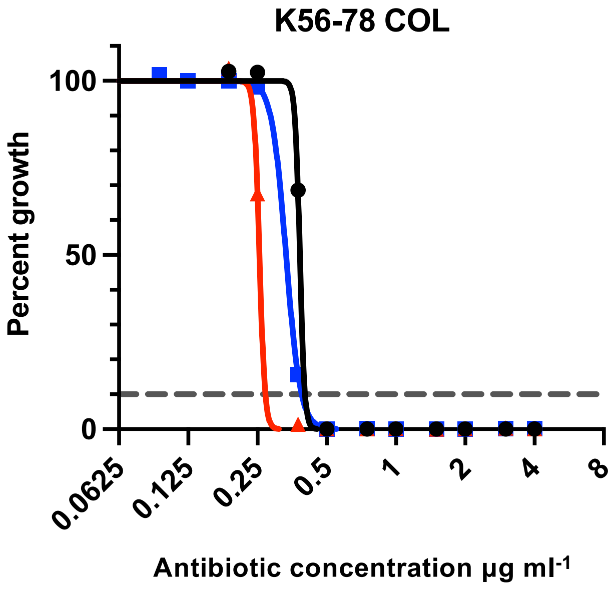

Supplementary figure 2 – Colistin:
Dose response curves denoting percent growth at given antibiotic concentrations for Ancestral WT (black circles), Ancestral ciprofloxacin resitant (red triangles) and Evolved ciprofloxacin restant (blue squares). Each strain background are separated into different graphs. Grey dashed line denotes the IC_{90}. Shown values are averages and CIP-R$_{evolved}$ is based on the average of the three evolved populations.

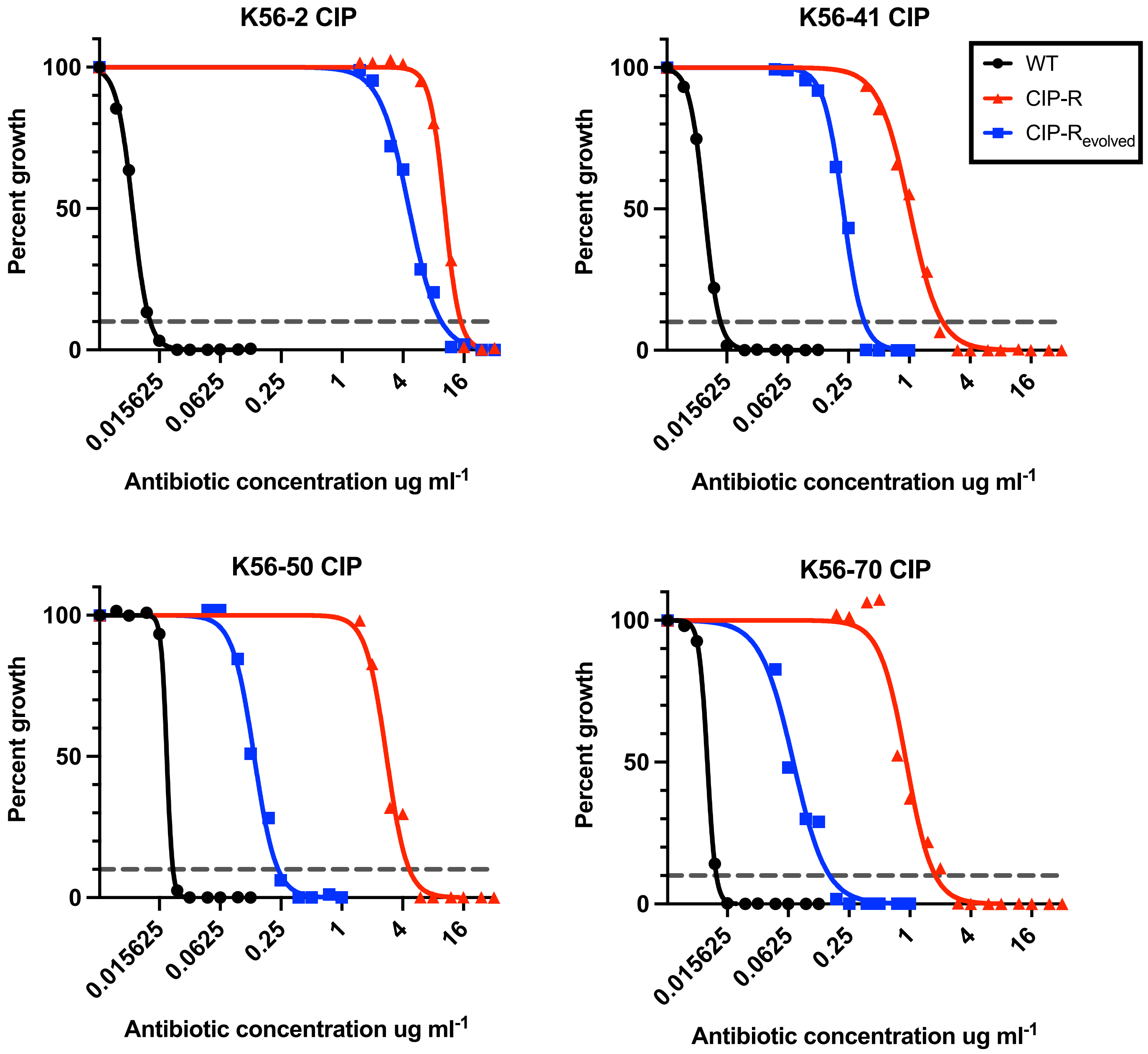

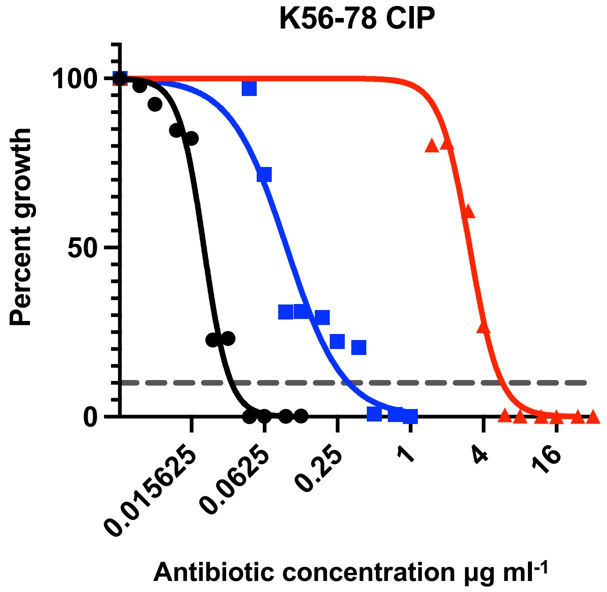

Supplementary figure 2 – Ciprofloxacin:
Dose response curves denoting percent growth at given antibiotic concentrations for Ancestral WT (black circles), Ancestral ciprofloxacin resitant (red triangles) and Evolved ciprofloxacin restant (blue squares). Each strain background are separated into different graphs. Grey dashed line denotes the IC_{90}. Shown values are averages and CIP-$R_{evolved}$ is based on the average of the three evolved populations.

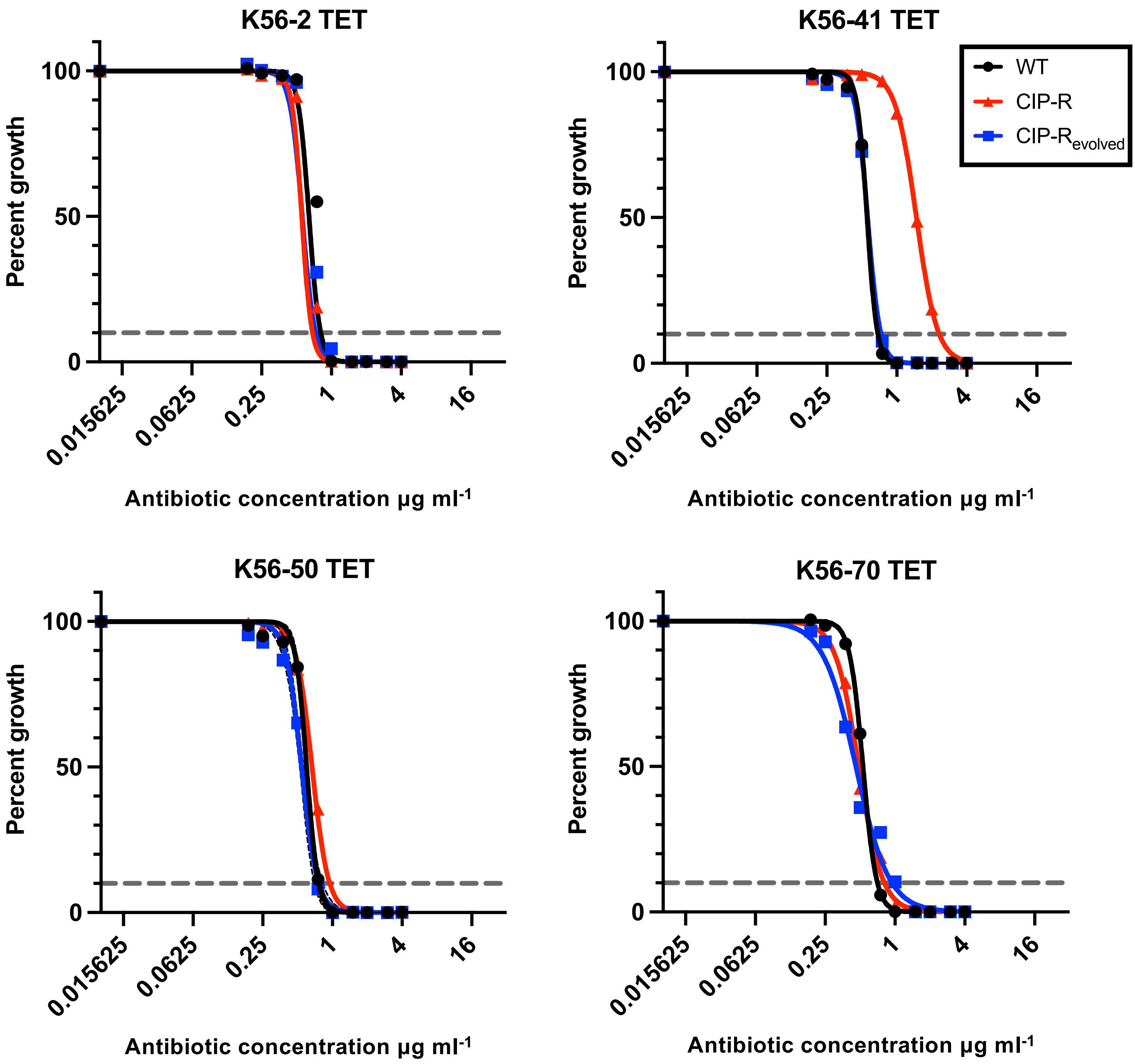

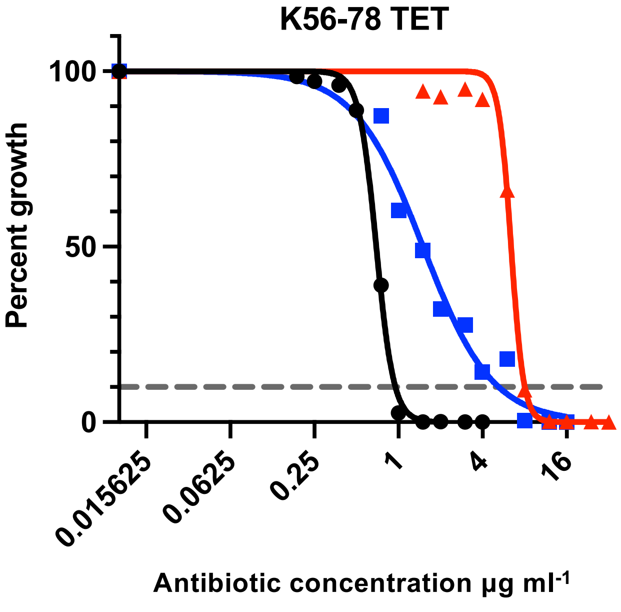

Supplementary figure 2 – Tetracycline:
Dose response curves denoting percent growth at given antibiotic concentrations for Ancestral WT (black circles), Ancestral ciprofloxacin resitant (red triangles) and Evolved ciprofloxacin restant (blue squares). Each strain background are separated into different graphs. Grey dashed line denotes the IC_{90}. Shown values are averages and CIP-R$_{evolved}$ is based on the average of the three evolved populations.

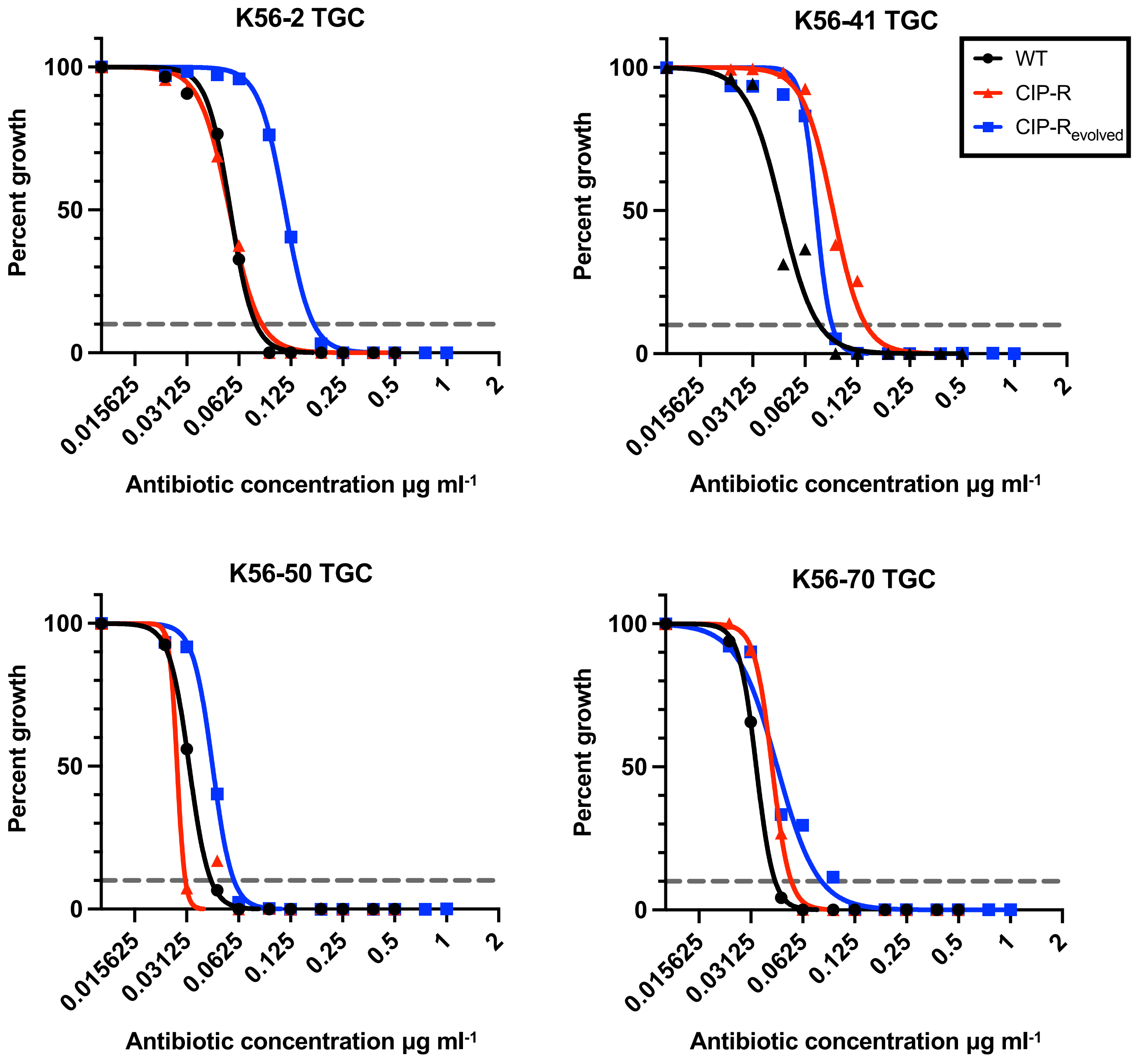

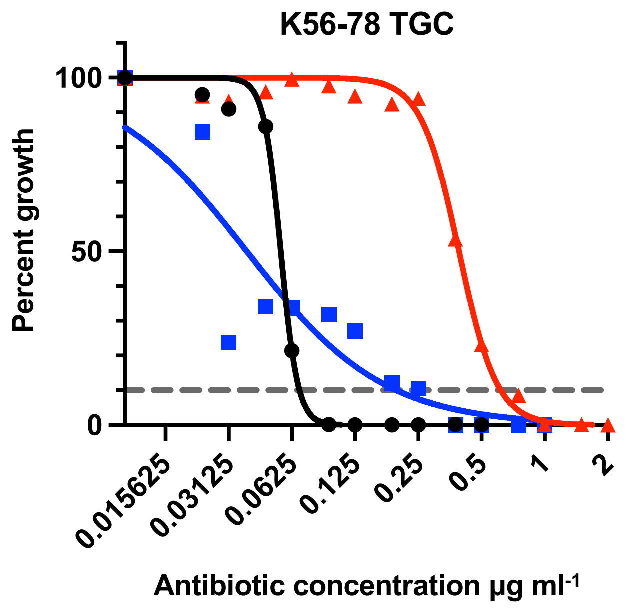

Supplementary figure 2 – Tigecycline: Dose response curves denoting percent growth at given antibiotic concentrations for Ancestral WT (black circles), Ancestral ciprofloxacin resitant (red triangles) and Evolved ciprofloxacin restant (blue squares). Each strain background are separated into different graphs. Grey dashed line denotes the IC_{90}. Shown values are averages and CIP-$R_{evolved}$ is based on the average of the three evolved populations.
